# Integrated Genomic Characterization Reveals Novel, Therapeutically Relevant Drug Targets in FGFR and EGFR Pathways in Sporadic Intrahepatic Cholangiocarcinoma

**DOI:** 10.1371/journal.pgen.1004135

**Published:** 2014-02-13

**Authors:** Mitesh J. Borad, Mia D. Champion, Jan B. Egan, Winnie S. Liang, Rafael Fonseca, Alan H. Bryce, Ann E. McCullough, Michael T. Barrett, Katherine Hunt, Maitray D. Patel, Scott W. Young, Joseph M. Collins, Alvin C. Silva, Rachel M. Condjella, Matthew Block, Robert R. McWilliams, Konstantinos N. Lazaridis, Eric W. Klee, Keith C. Bible, Pamela Harris, Gavin R. Oliver, Jaysheel D. Bhavsar, Asha A. Nair, Sumit Middha, Yan Asmann, Jean-Pierre Kocher, Kimberly Schahl, Benjamin R. Kipp, Emily G. Barr Fritcher, Angela Baker, Jessica Aldrich, Ahmet Kurdoglu, Tyler Izatt, Alexis Christoforides, Irene Cherni, Sara Nasser, Rebecca Reiman, Lori Phillips, Jackie McDonald, Jonathan Adkins, Stephen D. Mastrian, Pamela Placek, Aprill T. Watanabe, Janine LoBello, Haiyong Han, Daniel Von Hoff, David W. Craig, A. Keith Stewart, John D. Carpten

**Affiliations:** 1 Division of Hematology/Oncology Mayo Clinic, Scottsdale, Arizona, United States of America; 2 Mayo Clinic Cancer Center, Scottsdale, Arizona, United States of America; 3 Center for Individualized Medicine, Mayo Clinic, Rochester, Minnesota, United States of America; 4 Department of Biomedical Statistics and Informatics, Mayo Clinic, Scottsdale, Arizona, United States of America; 5 Translational Genomics Research Institute, Phoenix, Arizona, United States of America; 6 Department of Pathology, Mayo Clinic, Scottsdale, Arizona, United States of America; 7 Department of Radiology, Mayo Clinic, Scottsdale, Arizona, United States of America; 8 Mayo Clinic Cancer Center, Rochester, Minnesota, United States of America; 9 Investigational Drug Branch, National Cancer Institute, Rockville, Maryland, United States of America; 10 Department of Laboratory Medicine and Pathology, Mayo Clinic, Rochester, Minnesota, United States of America; University of Washington, United States of America

## Abstract

Advanced cholangiocarcinoma continues to harbor a difficult prognosis and therapeutic options have been limited. During the course of a clinical trial of whole genomic sequencing seeking druggable targets, we examined six patients with advanced cholangiocarcinoma. Integrated genome-wide and whole transcriptome sequence analyses were performed on tumors from six patients with advanced, sporadic intrahepatic cholangiocarcinoma (SIC) to identify potential therapeutically actionable events. Among the somatic events captured in our analysis, we uncovered two novel therapeutically relevant genomic contexts that when acted upon, resulted in preliminary evidence of anti-tumor activity. Genome-wide structural analysis of sequence data revealed recurrent translocation events involving the *FGFR2* locus in three of six assessed patients. These observations and supporting evidence triggered the use of FGFR inhibitors in these patients. In one example, preliminary anti-tumor activity of pazopanib (*in vitro* FGFR2 IC_50_≈350 nM) was noted in a patient with an *FGFR2-TACC3* fusion. After progression on pazopanib, the same patient also had stable disease on ponatinib, a pan-FGFR inhibitor (*in vitro*, FGFR2 IC_50_≈8 nM). In an independent non-FGFR2 translocation patient, exome and transcriptome analysis revealed an allele specific somatic nonsense mutation (E384X) in *ERRFI1*, a direct negative regulator of *EGFR* activation. Rapid and robust disease regression was noted in this *ERRFI1* inactivated tumor when treated with erlotinib, an EGFR kinase inhibitor. *FGFR2* fusions and *ERRFI* mutations may represent novel targets in sporadic intrahepatic cholangiocarcinoma and trials should be characterized in larger cohorts of patients with these aberrations.

## Author Summary

Cholangiocarcinoma is a cancer that affects the bile ducts. Unfortunately, many patients diagnosed with cholangiocarcinoma have disease that cannot be treated with surgery or has spread to other parts of the body, thus severely limiting treatment options. New advances in drug treatment have enabled treatment of these cancers with “targeted therapy” that exploits an error in the normal functioning of a tumor cell, compared to other cells in the body, thus allowing only tumor cells to be killed by the drug. We sought to identify changes in the genetic material of cholangiocarcinoma patient tumors in order to identify potential errors in cellular functioning by utilizing cutting edge genetic sequencing technology. We identified three patient tumors possessing an *FGFR2* gene that was aberrantly fused to another gene. Two of these patients were able to receive targeted therapy for FGFR2 with resulting tumor shrinkage. A fourth tumor contained an error in a gene that controls a very important cellular mechanism in cancer, termed epidermal growth factor pathway (EGFR). This patient received therapy targeting this mechanism and also demonstrated response to treatment. Thus, we have been able to utilize cutting edge technology with targeted drug treatment to personalize medical treatment for cancer in cholangiocarcinoma patients.

## Introduction

Biliary tract cancers (BTC) comprise malignant tumors of the intrahepatic and extrahepatic bile ducts. Known risk factors for BTC are the liver flukes *O. viverrini* and *C. sinensis* in high prevalence endemic regions in southeast Asia [1]–[3], as well as primary sclerosing cholangitis [4]–[7], Caroli's disease [8], hepatitis B and hepatitis C [9]–[14], obesity [13], hepatolithiasis [15], [16] and thorotrast contrast exposure [17], [18]. Surgical approaches such as resection and liver transplantation represent the only curative treatment approaches for BTC [19]. Unfortunately, most patients present with surgically unresectable and/or metastatic disease at diagnosis. Systemic therapy with gemcitabine and cisplatin has been established as the standard of care for patients with advanced disease, but is only palliative [20], emphasizing the imminent need for novel therapies.

Multiple studies have reported the presence of mutations/allelic loss of known cancer genes in BTC [21]–[39] and recently, a prevalence set of 46 patients was used to validate 15 of these genes including: *TP53*, *KRAS*, *CDKN2A* and *SMAD4* as well as *MLL3*, *ROBO2*, *RNF43*, *GNAS*, *PEG3*, *XIRP2*, *PTEN*, *RADIL*, *NCD80*, *LAMA2* and *PCDHA13*. Recent studies have also identified recurrent mutations in *IDH1* (codon 132) and *IDH2* (codons 140 and 172) with a prevalence of 22–23% associated with clear cell/poorly differentiated histology and intrahepatic primary [40], [41]. Fusions with oncogenic potential involving the kinase gene *ROS1* have been identified in patients with BTC with a prevalence of 8.7% in a recent study [42]. Less frequently, mutations in sporadic BTC have been reported in *EGFR* [43], [44], *BRAF* [45], *NRAS* [40], [46], *PIK3CA* [40], [46], [47], *APC* [40], *CTNNB1* [40], *AKT1* [40], *PTEN* [40], *ABCB4* [48], *ABCB11* [49], [50], and *CDH1* [51] as well as amplifications in *ERRB2* [52].

Recently, two independent studies reported the presence of FGFR fusions in cholangiocarcinoma; a single case with *FGFR2-AHCYL1* [53] as well as several cases identifying *FGFR2-BICC1* fusions [53], [54]. Arai et al. evaluated the presence of *FGFR2* fusions in a cohort of 102 cholangiocarcinoma patients observing that the fusions occurred exclusively in the intrahepatic cases with a prevalence of 13.6% [53]. Due to the presence of known dimerization motifs in the fusion partners, Wu et al. conducted mechanistic studies that demonstrated the i*n vitro* interaction of FGFR2-BICC1 and other fusions that was not observed in the presence of wildtype *FGFR2* [54]. Furthermore, overexpression of the *FGFR2-BICC1* and other selected fusions resulted in altered cell morphology and increased cell proliferation [54]. These data led to the conclusion that the fusion partners are facilitating oligomerization, resulting in FGFR kinase activation in tumors possessing FGFR fusions. In addition, *in vitro* and *in vivo* assessment of the sensitivity of cell lines containing an FGFR2 fusion to an FGFR inhibitor demonstrated sensitivity to treatment only in the fusion containing cells [53], [54], suggesting the presence of FGFR fusions may be a useful predictor of tumor response to FGFR inhibitors.

To comprehensively explore the genetic basis of sporadic intrahepatic cholangiocarcinoma (SIC), with emphasis on elucidation of therapeutically relevant targets, we performed integrated whole genome and whole transcriptome analyses on tumors from 6 patients with advanced, sporadic intrahepatic cholangiocarcinoma (SIC). Notably, recurrent fusions involving the oncogene *FGFR2* (n = 3) were identified. A patient whose tumor presented with an *FGFR2-MGEA5* fusion has demonstrated preliminary evidence of anti-tumor activity manifest as stable disease accompanied by CA19-9 reduction and tumor necrosis to ponatinib, a pan-FGFR inhibitor (*in vitro* FGFR1 IC_50_≈24 nM, FGFR2 IC_50_≈8 nM, FGFR3 IC_50_≈8 nM and FGFR4 IC_50_≈34 nM). In another patient whose tumor possessed an *FGFR2-TACC3* fusion, preliminary anti-tumor activity of pazopanib (*in vitro* FGFR2 IC_50_≈350 nM) was also noted. After progression on pazopanib, the same patient also responded to ponatinib and again demonstrated tumor shrinkage. Additionally, a non-FGFR fusion patient was found to have allele-specific preferential expression of a loss of function mutation in *ERRFI1*, a direct negative regulator of EGFR activation. Similarly, rapid and robust disease regression was noted in the patient with an *ERRFI1* mutant tumor when treated with erlotinib, an EGFR kinase inhibitor. Results suggest that these novel targets in the EGFR and FGFR pathways may be therapeutically relevant in patients with sporadic cholangiocarcinoma.

## Results

### Genomic landscape

**Figure 1:**
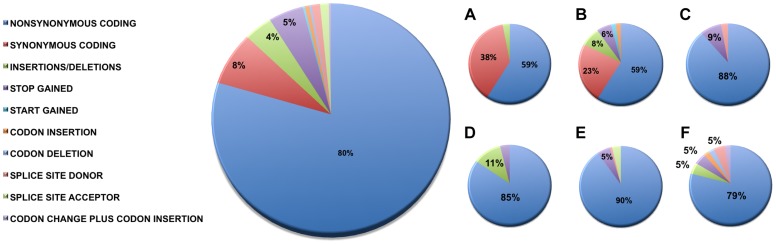
Sequence variation effects. Functional effects of high confidence sequence variations for all of the patients were identified as described in the Methods. The abundance of variations in each functional category is provided as percentages relative to the total number of high confidence variations and raw counts are provided in **Table 1**. For categories where the percentage was less than 5%, values are not shown. Summaries by individual patients are shown as follows: **A**) Patient 1, **B**) Patient 2, **C**) Patient 3, **D**) Patient 4, **E**) Patient 5, and **F**) Patient 6. Nonsynonymous single nucleotide variations were the predominant class in all of the patients. Two patients, Patients 1 and 2 also accumulated a high number of synonymous mutations in comparison to the other patients; Patient 5 carries the most stops gained likely contributing to a higher number of pseudogenes in comparison to the others; Patient 5 was also the only patient to carry several predicted high impact mutations that affect the splice site acceptor regions (light green, percentage <5%). In addition to the major functional classes summarized, Patient 6 also carried a codon change plus insertion variation.

**Figure 2:**
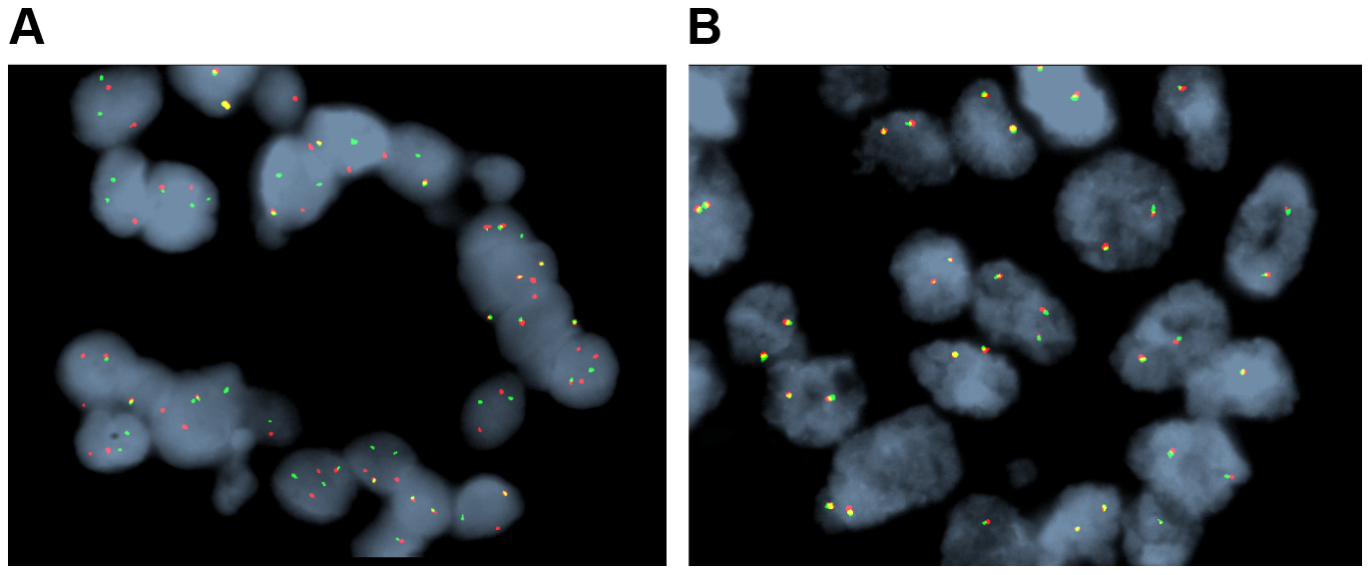
Representative fluorescent *in situ* hybridization (FISH) demonstrating the presence of FGFR2 fusion. A) Cholangiocarcinoma with FGFR2 rearrangement (distinct orange and green signals are present in most of the cells). B) Cholangiocarcinoma negative for FGFR2 rearrangement (orange and green signals remain fused).

We identified 327 somatic coding mutations, with an average of 55 mutations/tumor (range 34–112), within our cohort (**Table 1** **,** **Figure 1**). Nonsynonymous single nucleotide variations were the predominant class in all of the patients. Patients 1 and 2 accumulated a high number of synonymous mutations in comparison to the other patients. Patient 5 carried the most stops gained likely contributing to a higher number of pseudogenes in comparison to the others and was also the only patient to carry several predicted high impact mutations affecting splice site acceptor regions (**Figure 1**, light green, percentage <5%). In addition, patient 6 also carried a codon change plus insertion variation. Sequencing statistics are provided in **Table 2**. Genes with mutations in more than one case included *CSPG4* (n = 2), *GRIN3A* (n = 2) and *PLXBN3* (n = 2) (**Table S1**); with half of these predicted to be potentially damaging by SIFT [55], Polyphen [56], Mutation Assessor [57] and Mutation Taster [58]. While there was overlap in the somatic landscape of SIC with liver-fluke associated cholangiocarcinoma, hepatocellular cancer and pancreatic cancer, most of the aberrations detected in our study were distinct (**Table 3**). More importantly, using previously published methods [59], we identified molecular fusions involving *FGFR2* that were felt to be therapeutically relevant in 3 patients. Additionally, these fusions were validated with a break apart Fluorescent *In situ* Hybridization (FISH) assay (**Figure 2**). Notably, the patients who did not harbor the *FGFR2* fusions were negative using the same assay. Two of the three patients with *FGFR2* fusions (Patients 4 and 6) were treated with FGFR inhibitors while the third patient (Patient 5), experienced clinical decline prior to the availability of results and as such did not receive any further therapy. Furthermore, overexpression of an SNV in *ERRFI1* (E384X), a negative regulator of EGFR, was detected in a non-FGFR2 translocation patient's tumor. Taken together, our results constitute important therapeutically actionable alterations in patients with advanced SIC (**Text S1**).

**Table 1:** Summary of mutation type by patient.

	Patient 1	Patient 2	Patient 3	Patient 4	Patient 5	Patient 6
Nonsynonymous coding	20	30	31	44	101	34
Synonymous coding	13	12	0	0	0	0
Insertions/deletions	1	4	0	6	0	2
Stop gained	0	3	3	2	6	2
Start gained	0	1	0	0	0	0
Codon insertion	0	1	0	0	0	1
Codon deletion	0	0	0	0	0	1
Splice site donor	0	0	1	0	1	2
Splice site acceptor	0	0	0	0	4	0
**Total**	**34**	**51**	**35**	**52**	**112**	**42**

**Table 2:** Sequencing metrics of 6 advanced, sporadic biliary tract cancer patients.

		Exome				Whole Genome			RNA Seq	
Patient	Tissue	Aligned Reads (Millions)	Mean Target Coverage	% Target Bases 10×	# of Functional Coding Variants	Aligned Reads (Millions)	Aligned Bases (Billions)	Physical Coverage	Aligned Reads (Millions)	Aligned Bases (Billions)
1	N	161	100	94%	-	266	22	37	-	-
	T	156	112	94%	21	228	18	35	100	8.1
2	N	176	74	94%	-	179	14	5	-	-
	T	202	81	94%	34	370	30	10	341	26
3	N	226	110	58%	-	296	24	50	163	13
	T	195	92	58%	52	321	26	50	101	8.1
4	N	167	80	95%	-	317	26	42	-	-
	T	202	93	96%	52	163	13	12	264	20
5	N	257	146	96%	-	335	27	51	-	-
	T	133	78	93%	250	349	28	39	401	31
6	N	350	243	92%	-	-	-	-	-	-
	T	340	245	92%	43	-	-	-	713	31
Liver Control	-	-	-	-	-	-	-	-	118	9.6

N = Normal, T = Tumor.

**Table 3:** Comparison of mutation frequency in cholangiocarcinoma, pancreatic and liver cancers.

Gene	Non-liver fluke CCA (n = 6)	Liver fluke associated CCA [111] (n = 54)	CCA [40] (n = 62)	PDAC [112] (n = 142)	HCC [113] (n = 149)
*AKT1*	0%	0%	1.6%	0%	0%
*APC*	0%	0%	0%	0%	1.3%
*ARID2*	0%	0%	NA	2.1%	6.0%
*BAP1*	16.7%	0%	NA	0%	0%
*BRAF*	0%	0%	1.6%	0.7%	0%
*CDKN2A*	0%	5.6%	NA	2.4%	7.4%
*CSPG4*	33.3%	0%	NA	0%	0.7%
*CTNNB1*	0%	0%	NA	0%	34.9%
*DMXL1*	0%	0%	NA	0%	0%
*EGFR*	0%	0%	0%	0%	0%
*ERRFI1*	16.7%	0%	NA	0%	0.7%
*FLT3*	0%	0%	0%	0%	0%
*GNAS*	0%	9.3%	NA	0.7%	0%
*GRIN3A*	33.3%	0%	NA	0%	0%
*IDH1*	0%	0%	13%	0%	0%
*IDH2*	16.7%	0%	2%	0%	0%
*JAK2*	0%	0%	0%	0%	0%
*KIT*	0%	0%	0%	0%	0%
*KRAS*	0%	16.7%	NA	66.2%	1.3%
*LAMA2*	16.7%	3.7%	NA	0%	0%
*MLL3*	16.7%	14.8%	NA	4.9%	0%
*NDC80*	0%	3.7%	NA	0%	0%
*NLRP1*	16.7%	0%	NA	0%	0%
*NOTCH1*	16.7%	0%	0%	0%	0%
*NRAS*	16.7%	0%	3.2%	0%	0%
*PCDHA13*	16.7%	3.7%	NA	0.7%	0%
*PAK1*	16.7%	0%	NA	0%	0%
*PEG3*	0%	5.6%	NA	1.4%	0%
*PIK3CA*	0%	0%	0%	0%	1.3%
*PLXNB3*	33.3%	0%	NA	0%	0%
*PTEN*	0%	3.7%	2%	0%	0%
*PTK2*	16.7%	0%	NA	0%	0%
*RADIL*	0%	3.7%	NA	0%	0%
*RNF43*	0%	9.3%	NA	0%	0%
*ROBO2*	0%	9.3%	NA	1.4%	0%
*SMAD4*	0%	16.7%	NA	11.3%	0%
*TP53*	33.3%	44.4%	8%	23.2%	19.5%
*XIRP2*	0%	5.6%	NA	3.5%	0%

CCA, cholangiocarcinoma; PDAC, pancreatic ductal adenocarcinoma; HCC, hepatocellular carcinoma; NA, not assessed.

### Pathway analysis

**Figure 3:**
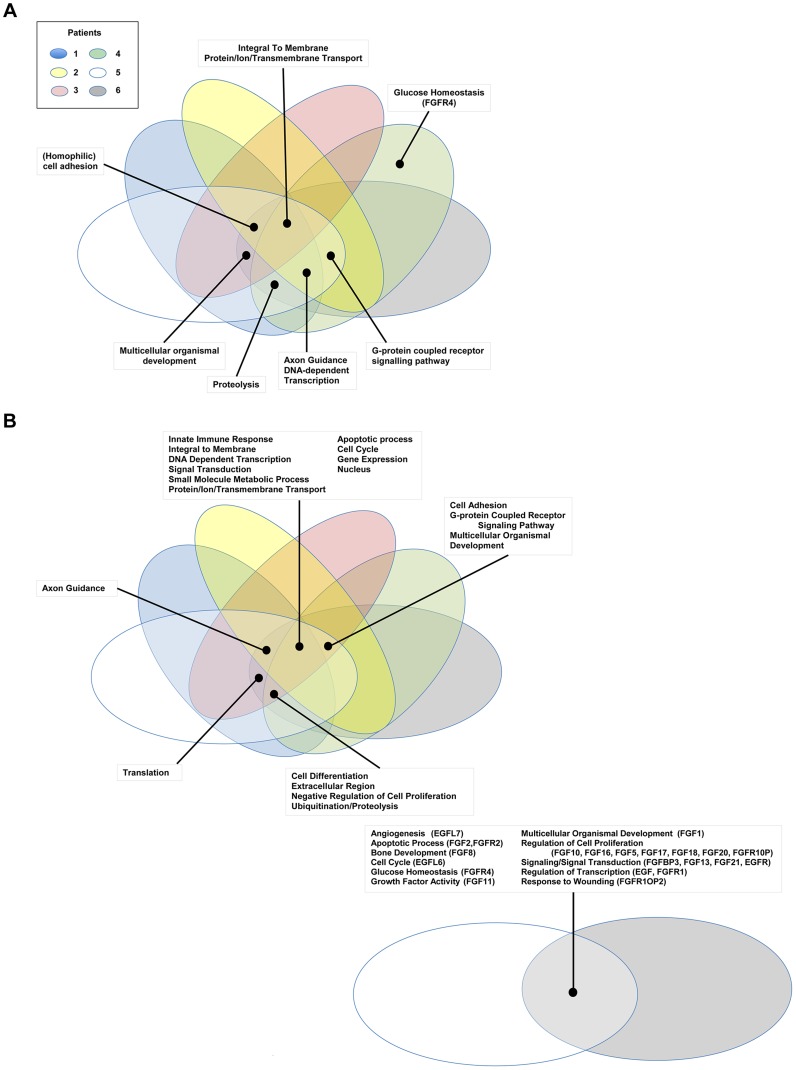
Gene Ontology pathway analysis. Genes carrying single nucleotide or frameshift variations, or aberrant in copy number were annotated and clustered by GO term functional classes, some of which are known to play a role in Cancer (**Tables S2 and S3**). Major classes for **A**) SNVs and **B**) CNVs are labeled in the figure. Proteins predicted to be integral to the membrane and involved in transport, as well as transcriptional regulators were among the most abundant class in all of the patients affected by small scale sequence variations and copy number variations. Variations specifically affecting the *EGFR* or *FGFR* gene families were prevalent in Patients 4, 5, and 6 and are highlighted in the figure with the gene name provided in parenthesis next to the pathway name.

Comparative pathway analysis of genes carrying small scale nucleotide variations (SsNVs) has implicated several major pathways, possibly interacting as a network, that are predicted to underlie disease in all of our studied biliary carcinoma patients. These shared pathways include EGFR, EPHB, PDGFR-beta, Netrin-mediated and Beta1 integrin mediated signaling pathways (**Figure 3** **and Tables S2 and S3**). Interestingly, most of these pathways have known roles in mediating epithelial-to-mesenchymal cell transitions, which occur frequently during development as well as tumorigenesis. Cell growth and motility is inherent to the successful progression of both biological processes. Studies of the nervous system and lung development have shown that Netrins act to inhibit FGF7 and FGF10 mediated growth or cell guidance [60]. In addition, Netrin-1 has a known role in mediating cell migration during pancreatic organogenesis [60]. Furthermore, Netrin-1 acts as a ligand for α3β1 and α6β4 integrins, both of which are involved in supporting adhesion of developing pancreatic epithelial cells with Netrin-1 although it is thought that α6β4 plays the principle role during this process [60]. Interestingly, α3β1 has been hypothesized to play a role during the process of angiogenesis, when chemoattractants and chemorepellents act to guide filopodia during migration [60]. The α3β1 integrin receptor may act together with additional pathways proposed to play a role during angiogenesis such as VEGF, PDGFR-beta [61], and EphrinB [62] as well as tumorigenesis [60]. Patients 3 and 4 also shared several genes acting in cadherin signaling pathways (**Tables S3, S4**), which are important for maintaining cell-cell adhesion and are known to be intimately integrated with EGFR and FGFR signaling pathways [63].

**Figure 4:**
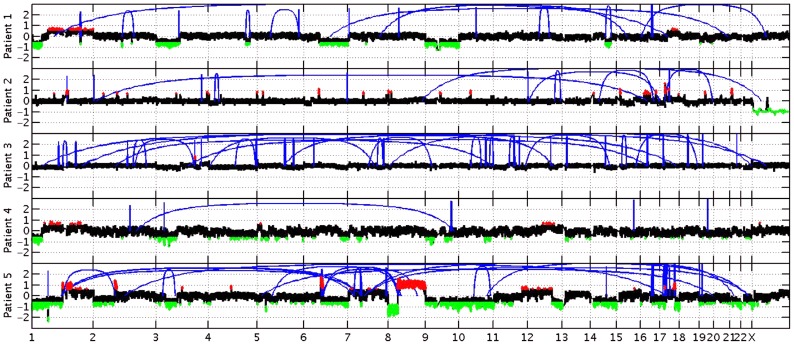
Copy number changes and structural rearrangements. Whole genome data was utilized to determine copy number alterations and structural rearrangements in the genome for Patients 1–5. WGS was not conducted for patient 6. Red indicates copy number gain, green copy number loss and blue lines indicate structural rearrangements. Significant variability between samples was observed for both copy number changes and structural rearrangements. Patient 5 presented with numerous copy number changes and structural rearrangements contrasting with patient 4 who had minimal structural rearrangements and much smaller regions of copy number changes. Patient 3 is characterized by a large number of structural rearrangements with almost no copy number alterations; in contrast, Patient 1 has a moderate number of structural variations, but has large regions of copy number gain and loss. Patient 2 has a moderate number of structural rearrangements with multiple focal amplifications across the genome.

In addition to the variations identified in genes acting in EGFR and/or FGFR signaling pathways, we also report multiple sSNVs and copy number variations (CNVs) (**Figure 4**) in genes such as *HDAC1*, *TP53*, *MDM2* and *AKT1*, acting in interaction networks or regulatory pathways involving the fusion partner genes in patients 5 (*BICC1*), and 6 (*TACC3*) (**Table 4**). Known mutations in *BICC1* have been shown to disrupt canonical Wnt signaling [64] and genes, such as *BCL9*, involved in this pathway are known to regulate a range of biological processes such as transcription and cell proliferation and carry variations in patient 5 (**Table 4**). *CSPG4*, a target that is being investigated for antibody-based immunotherapy in preclinical studies of triple negative breast cancer [65], is involved in the Wnt signaling pathway, and carries variations in both patients 1 and 2, however, it is not mutated in patient 5. TACC3 is known to mediate central spindle assembly and multiple genes including CDCA8, BUB1, and TACC1, belonging to the TACC3 interaction network exhibit aberrant copy number in patient 6 (**Table 4**). A recent study has also implicated TACC3 in EGF-mediated EMT when overexpressed [64], and we find that the *PLCG1*, *MAP2K1*, and *MAPK8* genes, which act in both FGFR and EGFR regulatory pathways, exhibit CNV in patient 6. We also note that the *DNAH5* gene encoding a dynein protein which is part of the microtubule-associated motor protein complex carries two G→C missense mutations in patient 6 (**Table S1**). Several genes carrying more than one variation in either the same patient or different patients also included genes with known roles similar to genes in FGFR/EGFR pathways including axon guidance, invasive growth, or cell differentiation (*NAV3*, *LAMC3*, *PLXNB3*, and *PTPRK*) (**Table S1**). In the case of patient 4, our studies suggest that the primary effect of the FGFR2-MGEA5 fusion is on FGFR2 related signaling, since changes in expression were observed in *FGF8* (p<0.05) and the genome of this patient also carries a 4-bp insertion (∧GTGT) in the *FGFR4* gene (**Table S1**).

**Table 4:** Stable fusion partner gene pathways.

Patients	Gene in Interaction or Regulatory Network	Small-scale Variation (sSNV)/Copy Number Variation (CNV)	Associated Network	Associated Pathway
4	FGFR4	ssNV	FGFR	glucose homeostasis
5	RAF1	CNV	EGFR/FGFR	axon guidance
5	RPS6KA5	CNV	FGFR	innate immune response
5	HGF	CNV	FGFR	mitosis
5	FRS2	CNV	FGFR	ventricular septum development
5	FGFR2	CNV	FGFR	apoptotic process
5	FGFR4	CNV	FGFR	glucose homeostasis
5	FGFR1OP2	CNV	FGFR	response to wounding
5	FGFR1	CNV	FGFR	transcription, DNA-dependent
5	ANTXR1	CNV	BICC1	actin cytoskeleton reorganization
5	ARL3	CNV	BICC1	cell cycle
5	NKX3-1	CNV	BICC1	multicellular organismal development
5	WIF1	CNV	BICC1	multicellular organismal development
5	AXIN2	CNV	BICC1	negative regulation of cell proliferation
5	SFRP1	CNV	BICC1	negative regulation of cell proliferation
5	HDAC1	CNV	BICC1	negative regulation of transcription from RNA polymerase II promoter
5	HNF1A	CNV	BICC1	positive regulation of transcription, DNA-dependent
5	NR5A2	CNV	BICC1	positive regulation of transcription, DNA-dependent
5	IPO13	CNV	BICC1	protein import into nucleus
5	MAP3K7	CNV	BICC1	transcription, DNA-dependent
5	SLC6A20	CNV	BICC1	transmembrane transport
5	BTRC	CNV	BICC1	ubiquitin-dependent protein catabolic process
5	BCL9	CNV	BICC1	Wnt receptor signaling pathway
5	TP53	ssNV	BICC1	transcription, DNA-dependent
6	PLCG1	CNV	EGFR/FGFR	axon guidance
6	MAP2K1	CNV	EGFR/FGFR	innate immune response
6	MAPK8	CNV	EGFR/FGFR	peptidyl-threonine phosphorylation
6	GAB1	CNV	FGFR	heart development
6	ATF2	CNV	FGFR	innate immune response
6	MAPKAPK2	CNV	FGFR	innate immune response
6	RPS6KA5	CNV	FGFR	innate immune response
6	HGF	CNV	FGFR	mitosis
6	FRS2	CNV	FGFR	ventricular septum development
6	FGF2	CNV	FGFR	apoptotic process
6	FGFR2	CNV	FGFR	apoptotic process
6	FGFR4	CNV	FGFR	glucose homeostasis
6	FGF17	CNV	FGFR	positive regulation of cell proliferation
6	FGF18	CNV	FGFR	positive regulation of cell proliferation
6	FGF20	CNV	FGFR	positive regulation of cell proliferation
6	FGFR1OP	CNV	FGFR	positive regulation of cell proliferation
6	FGFR1	CNV	FGFR	transcription, DNA-dependent
6	MDM2	CNV	TACC3	protein ubiquitination
6	E2F2	CNV	TACC3	apoptotic process
6	GADD45A	CNV	TACC3	apoptotic process
6	HMGB2	CNV	TACC3	apoptotic process
6	RHOA	CNV	TACC3	axon guidance
6	PEBP1	CNV	TACC3	brain development
6	EVI5	CNV	TACC3	cell cycle
6	CDCA8	CNV	TACC3	cell division
6	CKAP5	CNV	TACC3	cell division
6	PPP1CC	CNV	TACC3	cell division
6	BUB1	CNV	TACC3	cell proliferation
6	GTSE1	CNV	TACC3	DNA damage response, signal transduction by p53 class mediator resulting in cell cycle arrest
6	TACC1	CNV	TACC3	microtubule cytoskeleton organization
6	KIF20A	CNV	TACC3	microtubule-based movement
6	KIF2C	CNV	TACC3	microtubule-based movement
6	NCAPH	CNV	TACC3	mitosis
6	NSUN2	CNV	TACC3	mitosis
6	AKAP9	CNV	TACC3	mitotic cell cycle
6	KIF23	CNV	TACC3	mitotic cell cycle
6	MCM5	CNV	TACC3	mitotic cell cycle
6	NPM1	CNV	TACC3	negative regulation of cell proliferation
6	CBX5	CNV	TACC3	negative regulation of transcription, DNA-dependent
6	MKI67	CNV	TACC3	organ regeneration
6	AURKAIP1	CNV	TACC3	positive regulation of proteolysis
6	AKT1	CNV	TACC3	protein ubiquitination
6	BRCA1	CNV	TACC3	protein ubiquitination
6	KLHL13	CNV	TACC3	protein ubiquitination
6	KLHL9	CNV	TACC3	protein ubiquitination
6	TTF2	CNV	TACC3	regulation of transcription, DNA-dependent
6	RACGAP1	CNV	TACC3	signal transduction
6	TDRD7	CNV	TACC3	spermatogenesis
6	PRKACA	CNV	TACC3	transmembrane transport

### FGFR2-MGEA5 as a putative therapeutic target

**Figure 5:**
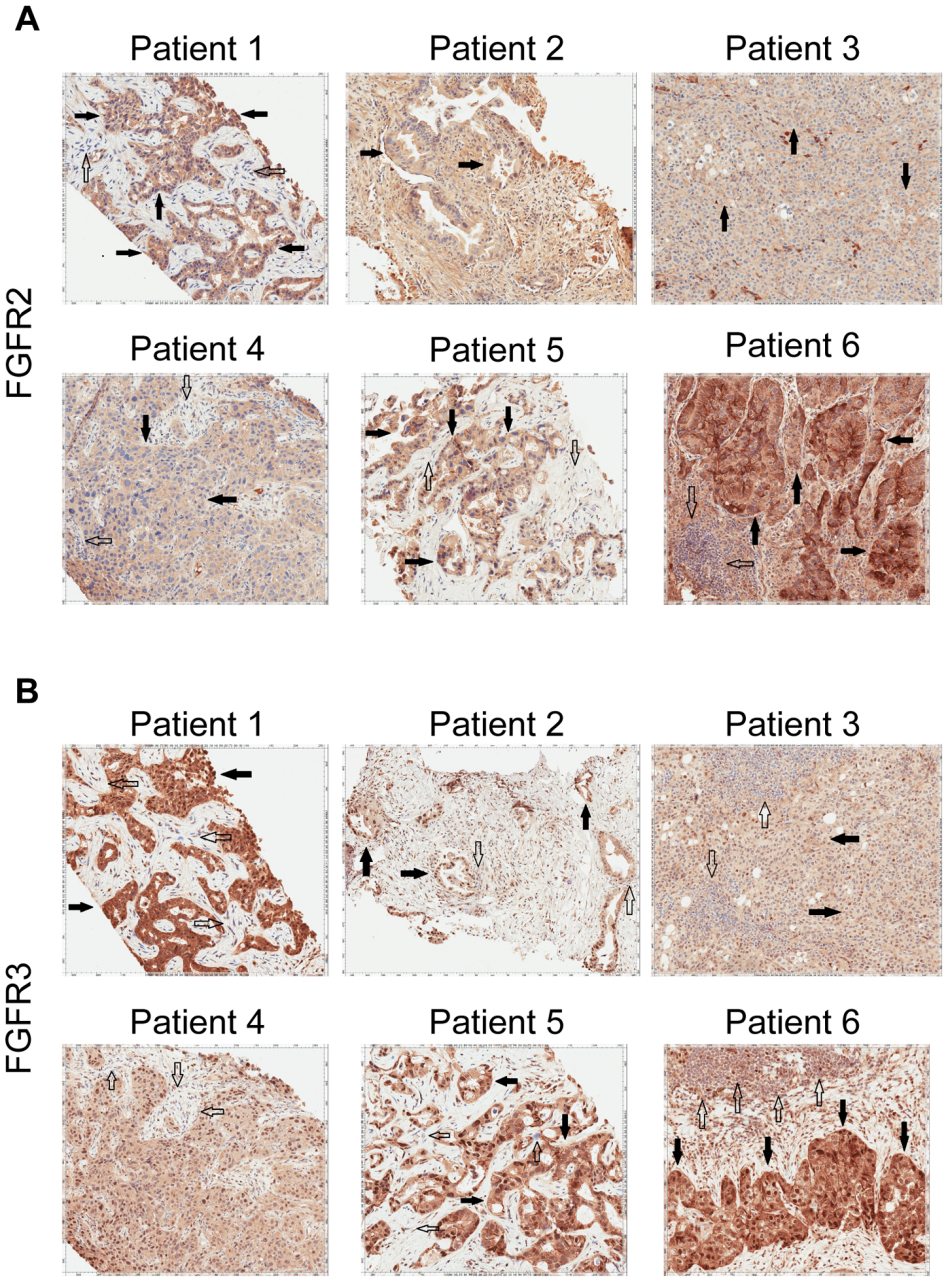
Immunohistochemistry demonstrating FGFR2 and FGFR3 expression. **A**) Tumor stained with FGFR2 antibody. Patient 1 demonstrates moderate cytoplasmic positivity (solid arrows); background fibro-inflammatory tissue is negative (empty arrows). Patient 2 demonstrates moderate cytoplasmic expression for FGFR2; tumor nuclei are negative. Patient 3 demonstrates tumor cells with negative nuclear and weak cytoplasmic expression of FGFR2 (solid arrows) with cells demonstrating moderate basolateral or complete membranous staining as well. Patient 4 demonstrates weak/moderate cytoplasmic positivity with multi-focal weak/moderate membranous expression (solid arrows); background fibro-inflammatory tissue demonstrates negative/weak staining (empty arrows). Patient 5 demonstrates weak/moderate cytoplasmic positivity with multi-focal moderate/strong membranous expression (solid arrows); background fibro-inflammatory tissue is negative (empty arrows). Patient 6 demonstrates moderate/strong cytoplasmic positivity (solid arrows); background lymphocytes are negative (empty arrows). **B**) Tumor stained with FGFR3 antibody. Patient 1 demonstrates strong cytoplasmic positivity, variable nuclear expression and occasional moderate/strong membranous expression (solid arrows); background fibrous tissue is negative (empty arrows). Patient 2 demonstrates negatively staining background neutrophils (focally intraepithelial-far right) (empty arrows) and tumor cells with strong nuclear expression and moderate cytoplasmic positivity (solid arrows). Patient 3 demonstrates negatively staining background inflammation (empty arrows) and tumor cells with weak nuclear expression and moderate cytoplasmic positivity (solid arrows). Patient 4 demonstrates weak/moderate cytoplasmic positivity and variable nuclear expression; background fibro-inflammatory tissue demonstrates negative/weak positivity (empty arrows). Patient 5 demonstrates moderate cytoplasmic positivity, variable nuclear expression and strong multi-focal membranous expression (solid arrows); background fibrous tissue is negative. Patient 6 demonstrates diffuse/moderate/strong cytoplasmic and membranous positivity and variable nuclear expression (solid arrows); background lymphocytes are negative (empty arrows).

**Figure 6:**
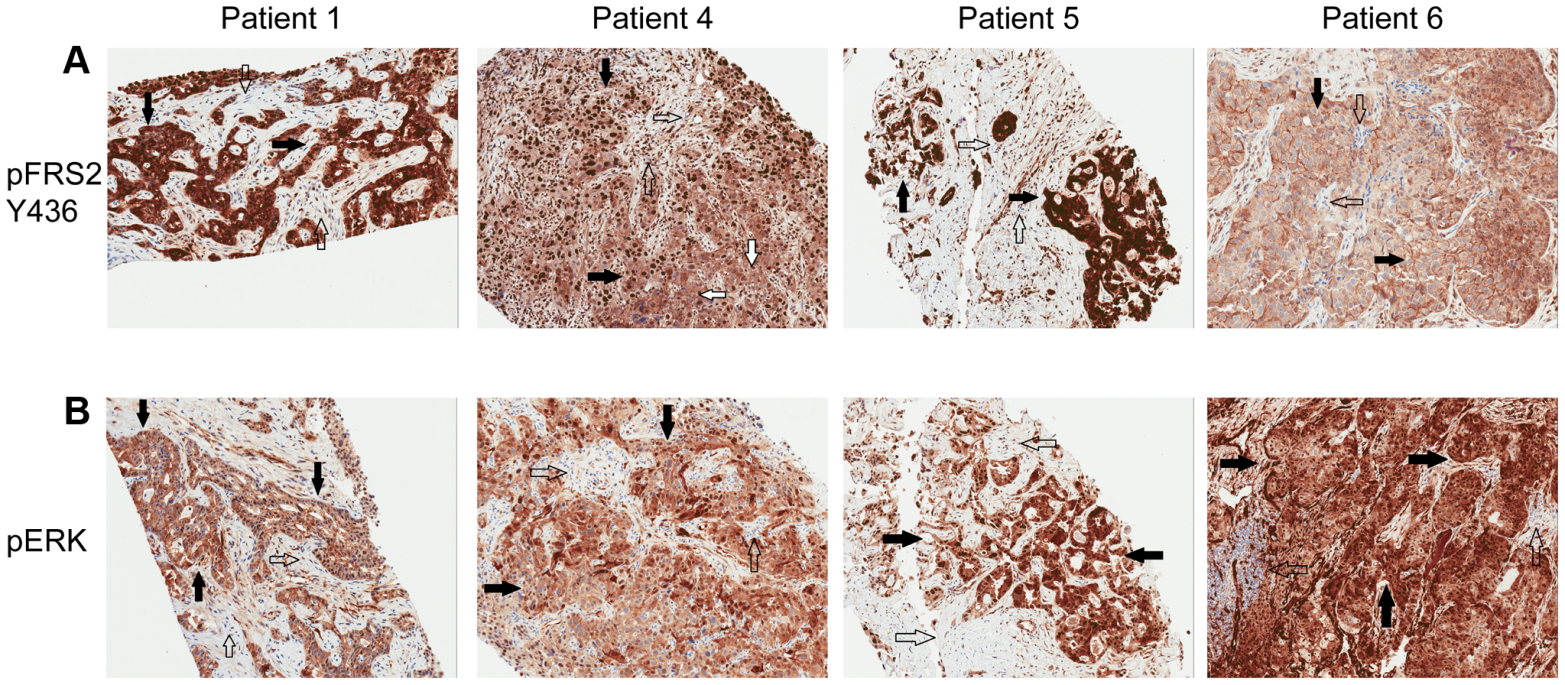
Immunohistochemistry demonstrating pFRS2 Y436, and pERK expression in Patients 1, 4, 5 and 6. **A**) Tumor stained with pFRS2 Y436 antibody. Patient 1 tumor cells demonstrating both strong cytoplasmic and nuclear expression of pFRS2 (solid arrows); background fibrous stroma is negative (empty arrows). Patient 4 tumor cells show strong nuclear expression and moderate to strong cytoplasmic positivity (solid arrows); occasional background fibrous stromal cells are negative for pFRS2 (empty arrows) and scattered tumor cells show basolateral/membranous staining as well (white arrows). Patient 5 tumor cells show intensely strong expression in both nuclei and cytoplasm (solid arrows); scattered background fibrous stromal cells are negative (empty arrows). Patient 6 tumor cells show negative nuclear expression of pFRS2, moderate cytoplasmic expression and basolateral or membranous expression of varying intensity (solid arrows); background fibrous stromal cells are negative (empty arrows). **B**) Tumor stained with pERK(MAPK) antibody. Patient 1 demonstrates negative/weak fibrous stroma (empty arrows) and tumor cells with negative nuclei and moderate to strong cytoplasmic expression (solid arrows). Patient 4 demonstrates negative inflammatory background (empty arrows) tumor cells with variable negative to strong nuclear expression and moderate to strong cytoplasmic positivity (solid arrows). Patient 5 demonstrates negative/weak fibrous stroma (empty arrows) and tumor cells with strong nuclear and cytoplasmic expression (solid arrows). Patient 6 demonstrates negative background lymphocytes/mononuclear inflammatory cells (empty arrows) and tumor cells with strong nuclear and cytoplasmic expression (solid arrows).

Patient 4 is a 62 year-old white female found to have a left-sided intrahepatic mass with satellite lesions, with metastasis to regional lymph nodes (**Table 5**). A biopsy of the liver mass revealed the presence of a poorly differentiated adenocarcinoma that was consistent with intrahepatic cholangiocarcinoma (CK7^+^, CEA^+^, CK20^+^, Hep-par 1^−^, TTF-1^−^) (**Table 6**). She received gemcitabine and cisplatin and obtained clinical benefit in the form of stable disease for 6 months, followed by disease progression. She was re-treated with gemcitabine and capecitabine systemic therapy and attained stable disease for 6 months, followed by disease progression. A clinical trial of pegylated hyaluronidase (PEGPH20) produced only stable disease for 4 months, followed again by disease progression. At this juncture, she underwent a liver biopsy to obtain tissue for whole genome characterization of her tumor. She was found to have an *FGFR2-MGEA5* fusion (**Table 7** **,** **Figure 2**) and ponatinib monotherapy was pursued as salvage treatment. Evaluation of pre-treatment immunohistochemistry demonstrated increased expression of *FGFR2* and *FGFR3* (**Figure 5**) and Clinical Laboratory Improvement Amendments (CLIA) validation by quantitative PCR revealed increased expression of *FGFR3* (**Table S5**). In order to further validate the activation of the receptor, we conducted immunohistochemistry (IHC) of pFRS2 Y436 and pERK(MAPK) that revealed strong expression of pFRS2 Y436 and pERK (**Figure 6**), thus confirming activation of the receptor.

**Table 5:** Clinical characteristics of 6 advanced, sporadic biliary tract cancer patients.

	Patient 1	Patient 2	Patient 3	Patient 4	Patient 5	Patient 6
Age (years)	64	66	50	62	50	43
Gender	F	M	M	F	F	F
Location of Primary Tumor	Intrahepatic	Intrahepatic/Gallbladder	Intrahepatic	Intrahepatic	Intrahepatic	Intrahepatic
Stage	III	IV	IV	IV	IV	IV
CA19-9 (Units/ml)	WNL	1008	WNL	WNL*	N/A	56
Sites of Metastasis	N/A	Abdominal Lymph Nodes	Cervical,Thoracic, Abdominal, Pelvic Lymph Nodes	Abdominal, Pelvic Lymph Nodes, Liver	Liver, Lungs, Peritoneum	Lungs
Underlying Etiology	Unknown	Unknown	Unknown	Unknown	Unknown	Unknown
Liver fluke	No	No	No	No	No	No
Hepatitis B	Unknown	Unknown	Negative	Unknown	Unknown	Unknown
Hepatitis C	Unknown	Unknown	Negative	Unknown	Unknown	Unknown
Prior Surgical Resection	No	Yes	Yes	No	Yes	No
Prior Radiation Therapy	No	No	No	No	No	No
Systemic Chemotherapy	Gem/Cis	Gem/Cis, Capecitabine	Gem/Cis	Gem/Cis, Gem/Cape, PEGPH20	Gem/Cis, 5-FU/Carbo	Gem/Cis, FOLFOX, Pazopanib
Survival Status	Alive	Dead	Dead	Alive	Dead	Alive
Survival Duration from biopsy (months)	14.5+	8.8	9.0	9.3+	4.1	5.5+

F = female; M = male; WNL = Within Normal Limits; Gem/Cis = Gemcitabine and Cisplatin; Gem/Cape = Gemcitabine and Capecitabine; PEGPH20 = pegylated hyaluronidase; 5-FU/Carbo = 5-Fluorouracil and Carboplatin; FOLFOX – 5-FU, Leucovorin and Oxaliplatin,

* = WNL at baseline but 1408 U/ml prior to therapy and N/A = Not Available.

**Table 6:** Pathological characteristics of 6 advanced, sporadic biliary tract cancer patients.

	Patient 1	Patient 2	Patient 3	Patient 4	Patient 5	Patient 6
Grade/differentiation	Moderate	Moderate	Undifferentiated**	Poor	Moderate	Poor
Biopsy Procedure	U/S Guided Liver Biopsy	U/S Guided Liver Biopsy	Excisional Biopsy Lymph Node	U/S Guided Liver Biopsy	U/S Guided Liver Biopsy	Excisional Lung Biopsy
%Necrosis (aliquots)	5 (1)	0 (2)	0–35 (7)	0 (3)	0–5 (3)	0
%Tumor	50	10–20	25–75	0–20	40–50	30
%Stroma and normal elements	50	80–90	25–75	80–100	50–60	70
Histological Type	NST*	NST	NST	NST	NST	NST
Clear Cell Histology (Yes/No)	No	No	No	No	No	No

U/S = Ultrasound.

* NST: No special type.

** Rare gland formation with expression of cytokeratin, polyclonal CEA, and MOC-31.

All were adenocarcinomas of no special types and high grades as defined by the World Health Organization Classification of Tumors of the Digestive System (Lyon 2000). Degree of differentiation is based on the percentage of glands (defined as having visible lumens by visual estimate) as follow: 95% or more glands-well differentiated, 40–94% glands-moderately differentiated, 5–39% glands-poorly differentiated, <5% glands-undifferentiated.

**Table 7:** Fusion events.

	Gene1	Gene2	Gene1 break location	Gene2 break location	Predicted Reciprocal Translocation	Patient
Fusions	*FGFR2*	*MGEA5*	chr10:123243211	chr10:103552699	No	4
	*FGFR2*	*BICC1*	chr10:123237843	chr10:60380614	Yes	5
	*BICC1*	*FGFR2*	chr10:60272900	chr10:123237848	Yes	5
	*FGFR2*	*TACC3*	chr10:123243211	chr4:1741428	No	6

**Figure 7:**
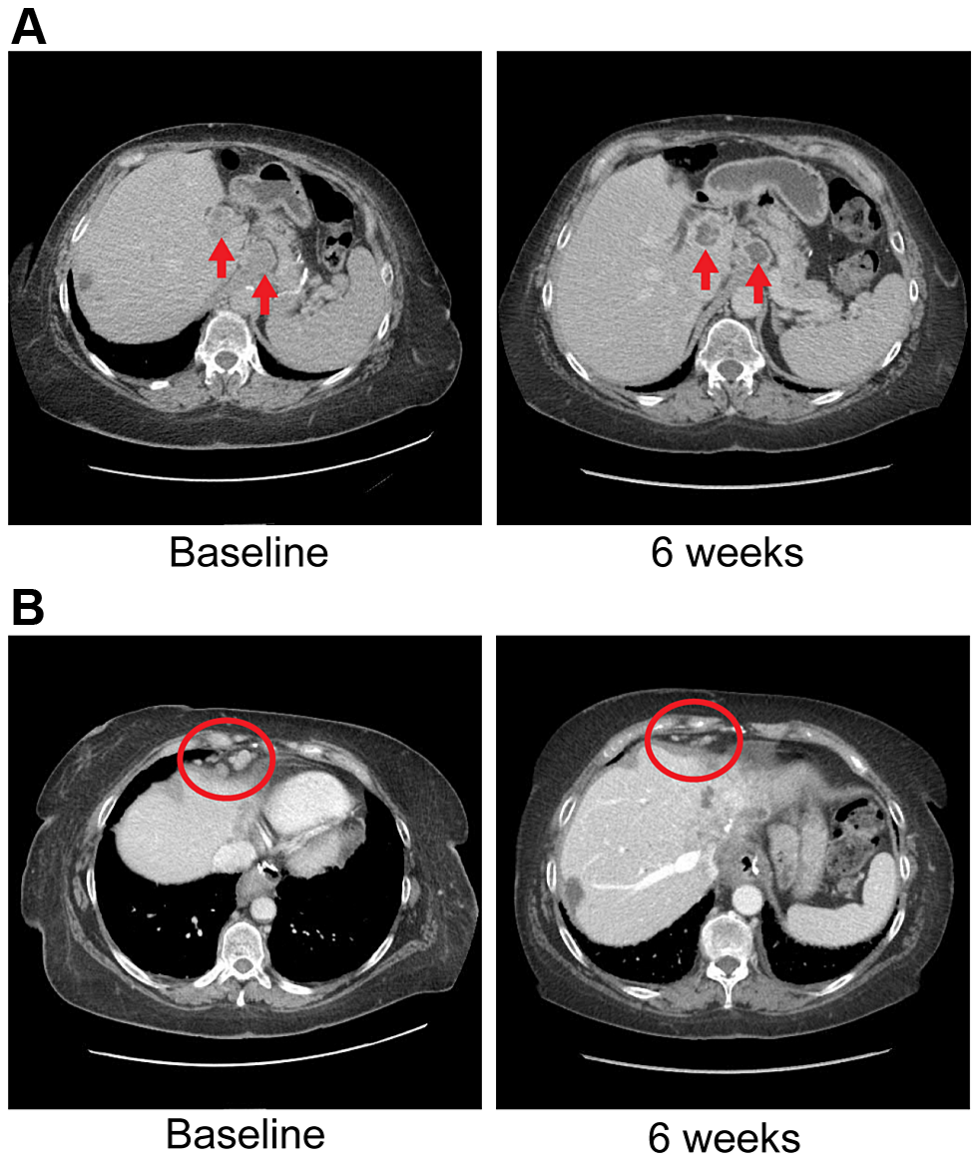
Anti-tumor activity in Patient 4 harboring an *FGFR2-MGEA5* fusion, to FGFR inhibitors. **A**) CT images of patient 4, whose tumor possessed an *FGFR2-MGEA5* fusion, at baseline and 6 weeks demonstrate central necrosis of a caudate liver lobe mass (left arrow), 2.6 cm at baseline and 6 weeks, and shrinkage of a metastatic supraceliac axis lymph node (right arrow), 3.1 cm and 2.9 cm at baseline and 6 weeks respectively. **B**) CT images of patient 4 showing shrinkage of metastatic lymph nodes involving the right cardiophrenic angle (red circles), 1.3 cm and 0.5 cm at baseline and 6 weeks respectively.

Ponatinib was initiated at 45 mg given orally on a daily schedule. Approximately 6 weeks after initiation of therapy she was noted to have anti-tumor activity that was characterized by central necrosis of a caudate liver lobe mass, shrinkage of metastatic lymph nodes involving the right cardiophrenic angle, central necrosis and shrinkage of a metastatic supraceliac axis lymph node (**Figure 7**) and reduction in CA 19-9 from 1408 U/ml to 142 U/ml. Per RECIST criteria, she exhibited stable disease with a 14% decrease in the sum of largest diameters but with tumor necrosis and reduction in the CA19-9 tumor marker (89.8%). While the evidence is preliminary in nature, it was felt that the combination of tumor shrinkage not meeting the RECIST criteria definition of partial response, tumor necrosis and reduction in CA19-9 constituted preliminary evidence of anti-tumor activity. She has experienced no treatment related toxicities thus far and remains on therapy of approximately 3.5 months duration thus far.

The *FGFR2* fusion partner observed in this patient, *MGEA5*, is an enzyme responsible for the removal of O-GlcNAc from proteins [66]. Interestingly, soft tissue tumors myxoinflammatory fibroblastic sarcoma (MIFS) and hemosiderotic fibrolipomatous tumor (HFLT) both share a translocation event resulting in rearrangements in *TGFBR3* and *MGEA5* [67], [68]. Associated with this translocation event is the upregulation of *NPM3* and *FGF8* [68], of which both genes are upregulated in this patient (fold change: *NPM3* = 6.17865, *FGF8* = 1.79769e+308). In breast cancer, grade III tumors had significantly lower *MGEA5* expression than grade I tumors with a trend of decreasing expression observed with increasing tumor grade [66]. In summary, MGEA5 may play an important role in carcinogenesis as an FGFR fusion partner.

### FGFR2-TACC3 as a putative therapeutic target

**Figure 8:**
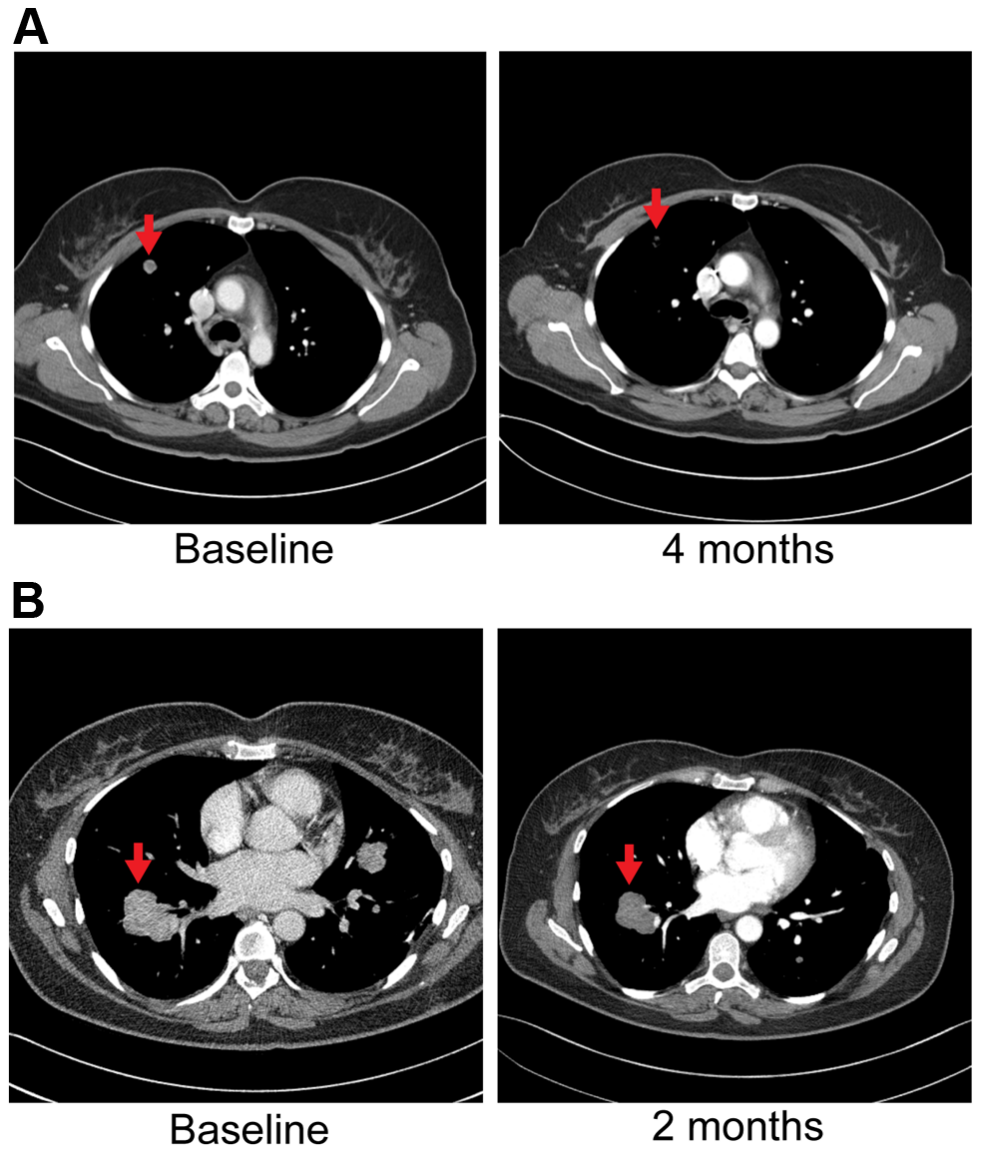
Anti-tumor activity in Patient 6, harboring an *FGFR2-TACC3* fusion, to FGFR inhibitors. **A**) CT images of patient 6, whose tumor possessed an *FGFR2-TACC3* fusion, at baseline and after four months of pazopanib demonstrate significant tumor shrinkage (red arrows), 10.8 mm and 3.1 mm respectively. **B**) CT images of patient 6 at baseline and two months demonstrate significant tumor shrinkage (red arrows), 41.1 mm and 39.4 mm respectively after subsequent ponatinib treatment, 45 mg/daily, was begun.

Patient 6 is a 43 year-old white female who underwent a right salpingo-oophorectomy and endometrial ablation in the context of a ruptured ovarian cyst (**Table 5**). Postoperatively she developed dyspnea and was found to have pulmonary nodules as well as a 5 cm left sided liver mass. Pathological evaluation of the liver mass was consistent with a moderately differentiated intrahepatic cholangiocarcinoma (CK7^+^, CK20^−^, TTF-1^−^) in the absence of any known risk factors (**Table 6**). She was treated systemically with gemcitabine and cisplatin and had stable disease for approximately 6 months, but was subsequently found to have disease progression. She was treated with FOLFOX for 7 months and again attained stable disease as best response to therapy but eventually experienced disease progression. Upon disease progression, she was enrolled on a clinical study with the multi-kinase inhibitor pazopanib that is FDA-approved for the treatment of advanced renal cancer or sarcoma – and fortuitously has nanomolar activity against FGFR2 (*in vitro* IC_50_ to FGFR2≈350 nM) [69]. Transcriptome analysis revealed the presence of an *FGFR2-TACC3* fusion (**Table 7** **,** **Figure 2**). Evaluation of post-pazopanib tissue by immunohistochemistry revealed increased expression of *FGFR2* and *FGFR3* (**Figure 5**) Further evaluation of phosphorylation of downstream targets FRS2 Y436, and ERK(MAPK) revealed strong expression of pERK and moderate expression of pFRS2 Y436 (**Figure 6**), confirming activation of the receptor. She had been treated with pazopanib 800 mg orally daily for 4 months and demonstrated tumor shrinkage, which in retrospect, was postulated to be secondary to the FGFR2 inhibitory activity of pazopanib (**Figure 8A**). By RECIST criteria v1.1, the patient had a partial response to therapy as evidenced by a 71% decrease in the sum of diameters. Subsequently, the same patient was treated with a dedicated pan-FGFR inhibitor, ponatinib, (45 mg daily orally; *in vitro* IC_50_ : FGFR1≈24 nM, FGFR2≈8 nM, FGFR3≈8 nM and FGFR4≈34 nM). She again attained minor tumor shrinkage (stable disease by RECIST criteria v1.1, decrease of 4% in sum of largest diameters) in multiple lesions after 2 months of therapy, despite undergoing a 50% dose reduction for abdominal pain felt to be related to drug (**Figure 8B**). She remains on therapy approximately 4 months since the initiation of ponatinib. As such, anti-tumor activity was obtained with two distinct FGFR inhibitors in the same patient.

The FGFR2 fusion partner observed in this patient's tumor, *TACC3*, is overexpressed in many tumor types with enhanced cell proliferation, migration, and transformation observed in cells overexpressing *TACC3* [70]. Furthermore regulation of ERK and PI3K/AKT by TACC3 may contribute in part to epithelial-mesenchymal transition (EMT) in cancer [70], a significant contributor to carcinogenesis. Interestingly, *TACC3* has been identified as a fusion partner to *FGFR3* in bladder cancer, squamous cell lung cancer, oral cancer, head and neck cancer and glioblastoma multiforme [54].

### ERRFI1 as a putative therapeutic target

Patient 3 was a 50 year-old white male who presented with fevers and night sweats (**Table 5**). He was found to have a 4 cm tumor in his liver determined to be a poorly differentiated intrahepatic cholangiocarcinoma (CK7^+^, CK20^−^, TTF1^−^, CD56^−^, synatophysin^−^, Hep-par 1^−^) with sclerotic features (**Table 6**). No overt risk factors for cholangiocarcinoma were identified. A left hepatectomy was undertaken three months later. In addition to the primary tumor in segment 4, limited resections of segments 6 and 8 were undertaken to remove two tumor nodules. He was soon noted to have increased hypermetabolism in the left lower cervical, upper mediastinal and abdomino-retroperitoneal lymph nodes related to metastatic disease from his cholangiocarcinoma. He was treated with gemcitabine and cisplatin for 9 months and obtained stable disease as his best response, followed by eventual progression. He received treatment with pegylated hyaluronidase (PEGPH20) in the setting of an investigational study for one month and had no response to therapy. A biopsy of a left supraclavicular lymph node was obtained two months prior to the initiation of PEGPH20 in the context of a clinical study employing whole genome analysis for putative therapeutic target selection.

Since our study goal was to identify potential therapeutically relevant events, the novel loss of function mutation in *ERRFI1* (E384X) detected in Patient 3's metastatic, recurrent/refractory SIC (**Table S1**) warranted additional examination. Specifically, the allelic fraction of the DNA mutation constituted only 11% of the sequencing reads, is consistent with tissue heterogeneity, and constituted 78% of the sequencing reads within the RNASeq data. Such allele specific expression of the mutated allele from the same tissue specimen suggests nearly complete loss of function of *ERRFI1* in this patient's tumor. Notably, the patient's tumor did not harbor any mutations or amplifications in other EGFR signaling members such as *EGFR* and *BRAF*.

**Figure 9:**
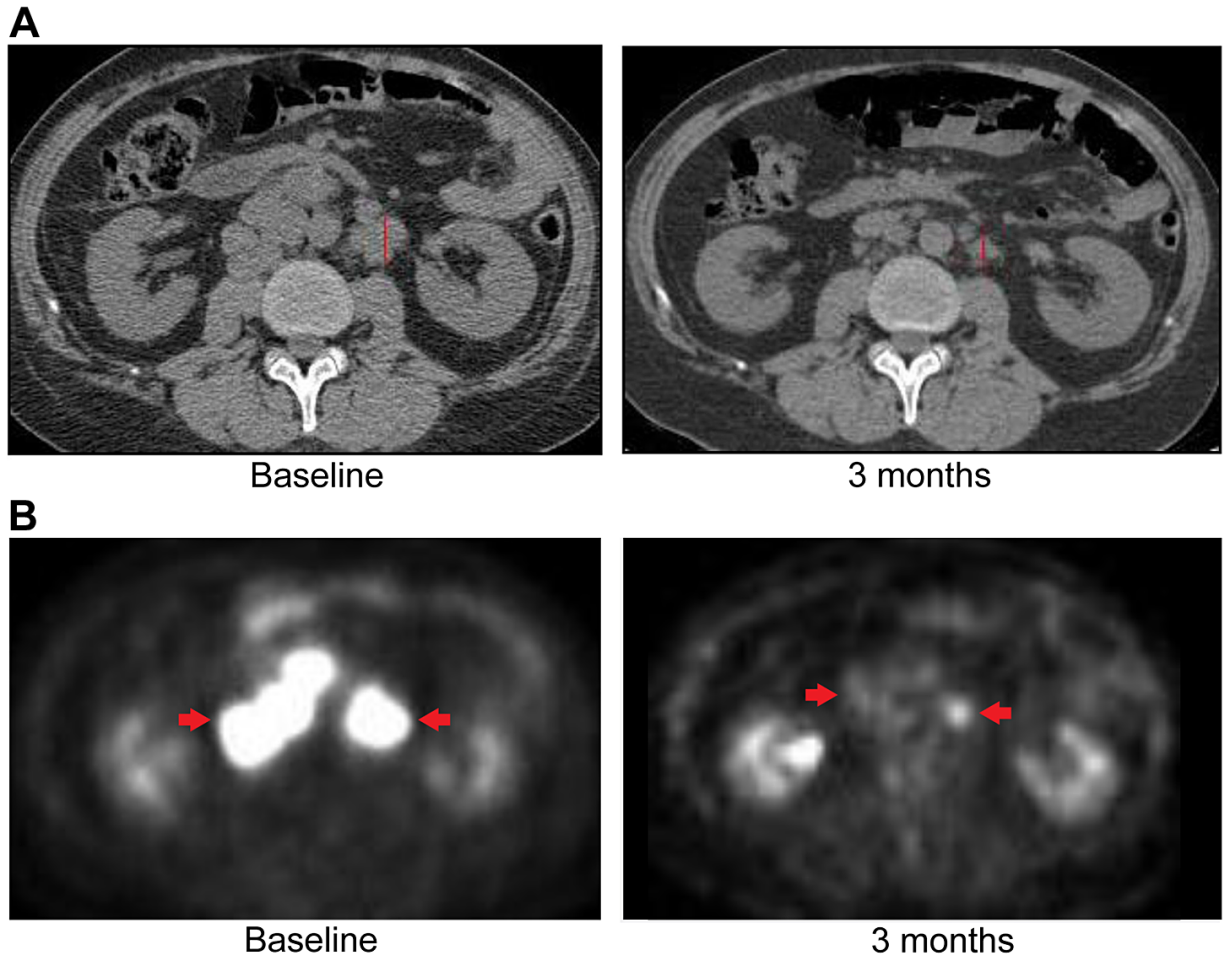
Anti-tumor activity of Patient 3, harboring an *ERRFI1* mutation, to erlotinib, an EGFR inhibitor. **A**) CT images of patient 3 at baseline and three months demonstrate significant tumor shrinkage (red marks). CT demonstrates right retroperitoneal lymph nodes decreasing from 7.6 cm to 2.9 cm and left retroperitoneal lymph nodes decreasing from 3.3 cm to 1.7 cm. **B**) PET images of patient 3 at baseline and three months demonstrate significant tumor shrinkage (red arrows). Hypermetabolic areas corresponding to right retroperitoneal lymph nodes demonstrate decrease from 8 cm longest diameter to imperceptible and left retroperitoneal lymph nodes decreasing from 4.2 cm to 1.4 cm. Both regions demonstrated significant reduction in metabolic activity.

**Figure 10:**
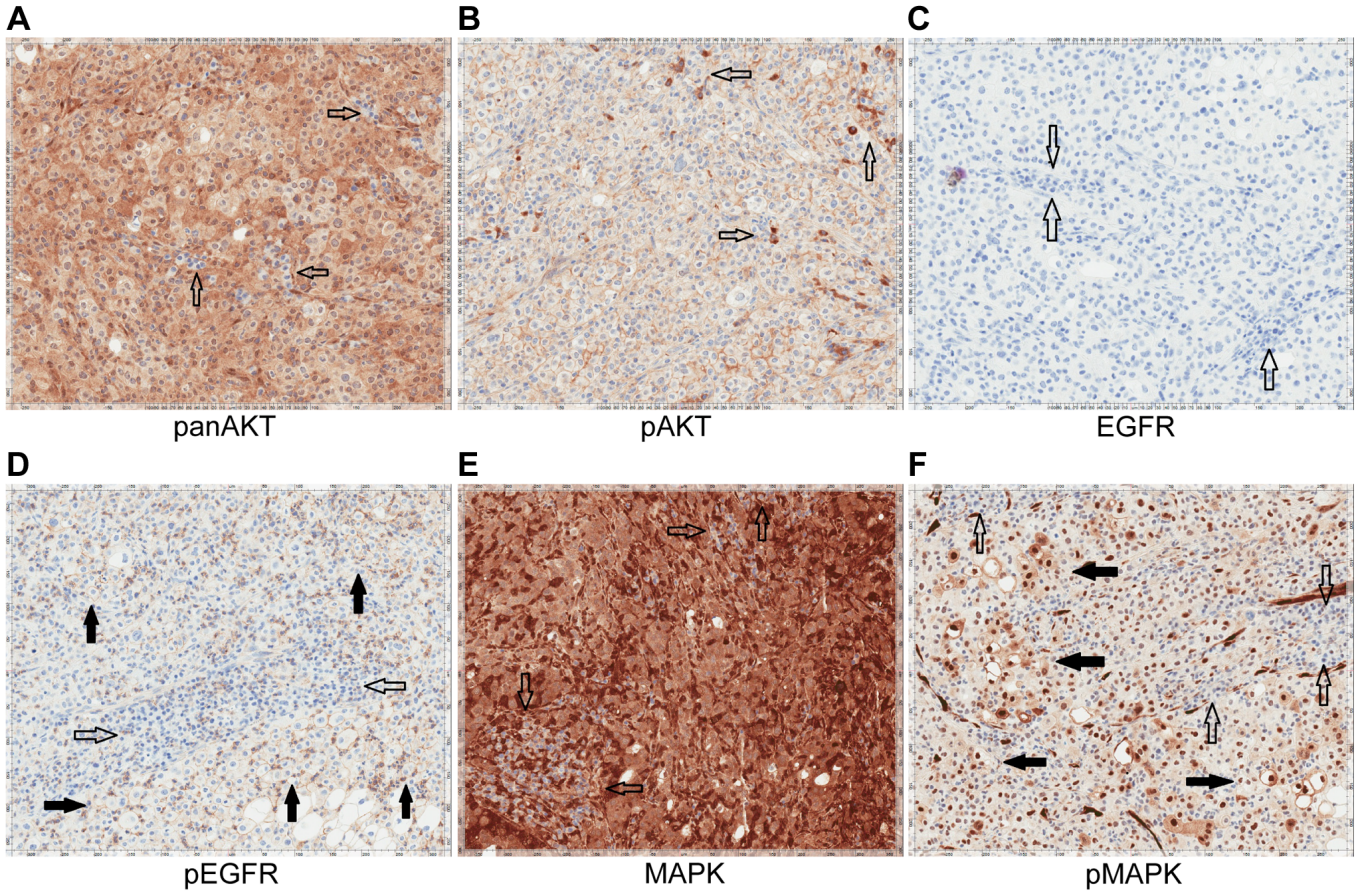
Immunohistochemistry of Patient 3's tumor demonstrating activation of the EGFR pathway. **A**) Tumor stained with panAKT demonstrating diffuse cytoplasmic positivity with negative background lymphocytes (empty arrows). **B**) Tumor stained with pAKT demonstrating diffuse membranous staining and negative cytoplasmic expression; scattered background inflammatory cells showing strong cytoplasmic staining (empty arrows). **C**) Tumor stained with EGFR. Tumor cells are EGFR negative with background lymphocytes also negative (empty arrows). **D**) Tumor stained with pEGFR showing membranous positivity (solid arrows) with negative background lymphocytes (empty arrows). **E**) Tumor stained with MAPK/ERK1/2 demonstrating moderate to strong cytoplasmic staining of total MAPK with negative background lymphocytes (empty arrows). **F**) Tumor stained with pMAPK/pERK demonstrating increased expression compared to the negative background lymphocytes (empty arrows).

Upon availability of CLIA validated sequencing data (**Table S5**), the patient was treated with erlotinib 150 mg orally/daily. After 3 months, RECIST v1.1 partial response evidenced by a decrease of 58% in the sum of largest diameters was observed (**Figure 9**). Evaluation of pretreatment tumor tissue by immunohistochemistry revealed increased expression of EGFR pathway members (**Figure 10**).

## Discussion

Integrated analysis of sporadic intrahepatic cholangiocarcinoma (SIC) genomic and transcriptomic data led to the discovery of *FGFR2* fusion products in three of six assessed patients (**Table 7** **,** **Figures 4** **and** **11**). Members of the FGFR family (*FGFR1-4*) have been associated with mutations, amplifications and translocation events with oncogenic potential [71]. FGFR fusions with oncogenic activity have been previously identified in bladder cancer (*FGFR3*) [72], lymphoma (*FGFR1* and *FGFR3*) [73], [74], acute myeloid leukemia (*FGFR1*) [75], multiple myeloma [76], myeloproliferative neoplasms [77], and most recently glioblastoma multiforme (*FGFR1* and *FGFR3*) [78]. *FGFR2*, *FGFR3* and *FGFR4* have been found to be overexpressed in *IDH1*/*IDH2* mutant biliary cancers [79], a context seen within Patient 1 in our study (**Tables S1 and S6,** **Figure 5**); although, no fusion events were depicted in these studies or in Patient 1.

**Figure 11:**
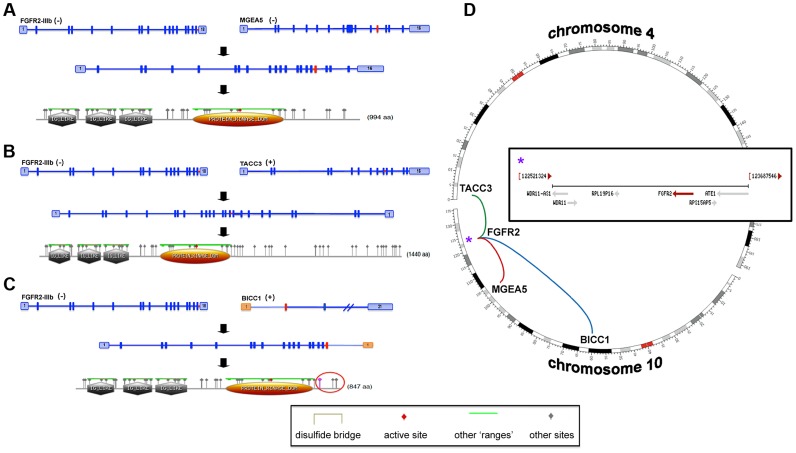
*FGFR2-IIIb* fusion events. Transcripts and hypothetical protein products are modeled to illustrate the potential functional impact of fusion events involving *FGFR2* (**A–C**). The identified fusion events involving *MGEA5* (patient 4) (**A**) and *BICC1* (patient 5, reciprocal event) (**C**) are chromosome 10 intrachromosomal (**D**). In addition, patient 6 carried an interchromosomal fusion event (**D**) involving *FGFR2* and *TACC3* (**B**). The *FGFR2* gene encodes for several isoforms with eleven representative transcripts and patients 4, 5, and 6 carry fusions involving the epithelial cell specific transcript isoform (*FGFR2*-IIIb). All identified fusion breakpoints are close in proximity and are predicted to occur within the last intron of the transcript and terminal to a known protein tyrosine kinase domain (**A–C**, gold domain). Predicted “Other” sites for all of the fusion protein models are the same and include the following: Casein kinase II phosphorylation sites, N-glycosylation sites, Protein kinase C phosphorylation sites, N-myristoylation sites, Tyrosine kinase phosphorylation sites, and cAMP-/cGMP-dependent protein kinase phosphorylation sites (**A–C**, grey triangle annotations). In all cases, fusions result in a predicted expansion of Casein kinase II phosphorylation and Protein kinase C phosphorylation sites. A protein product model is shown only for one of the reciprocal events involving the *FGFR2* and *BICC1* genes (*FGFR2*→*BICC1*, **C**). The fusion breakpoints of the reciprocal events effect Exons 1 and 2 of the BICC1 gene, which translates to a difference of a predicted phosphoserine site within the Casein kinase II phosphorylation region (**C**, purple triangle within red circle). The FGFR2 gene is located within a fragile site region (FRA10F) and is flanked by two ribosomal protein pseudogenes, RPS15AP5 and RPL19P16 (see D inset (*)), whose repetitive sequence content may also contribute to genomic instability at the *FGFR2* initiation site.

Although the gene partner fused to *FGFR2* was different for each patient (*MGEA5*, *BICC1* and *TACC3*), the breakpoints in *FGFR2* all occurred within the last intron distal to the last coding exon and terminal protein tyrosine kinase domain (**Figure 11**). All three fusions were validated at the DNA and/or RNA level (**Table 8**). Amongst these fusions, the *FGFR2-BICC1* fusion has recently been independently identified in SIC [53], [54]. For this particular fusion product we observed, and validated, the presence of two fusion isoforms (*FGFR2*-*BICC1* and *BICC1-FGFR2*). Interestingly, *BICC1* is a negative regulator of Wnt signaling [80] and when comparing expression of tumor and normal tissue we observed differentially expressed Wnt signaling genes, *APC* (fold change -4.75027), *GSK3B* (fold change -3.35309), and *CTNNB1* (fold change -1.73148), yet when the expression was compared to other cholangiocarincomas, no difference was observed.

**Table 8:** DNA and RNA validation of FGFR2 fusions in 3 patients with advanced sporadic biliary tract cancer.

Fusion	Annealing site	PCR input	Direction	Primer sequence
*FGFR2-MGEA5*	*FGFR2*	**gDNA**	F	5′-CTGACTATAACCACGTACCC-3′
	*MGEA5*	**gDNA**	R	5′-AGGGAGAAATTAAAGAACTTGG-3′
	*FGFR2*	**cDNA**	F	5′-TGATGATGAGGGACTGTTG-3′
	*MGEA5*	**cDNA**	R	5′-GAGTTCCTTGTCACCATTTG-3′
*FGFR2-BICC1*	*FGFR2*	**gDNA**	F	5′-GGCAGAAGAAGAAAGTTGG-3′
	*BICC1*	**gDNA**	R	5′-ACTACTGCAGTTTGTTCAAT-3′
	*FGFR2*	**cDNA**	F	5′-TGATGATGAGGGACTGTTG-3′
	*BICC1*	**cDNA**	R	5′-TGTGTGCTCACAGGAATAG-3′
*BICC1-FGFR2*	*BICC1*	**cDNA**	F	5′ CGTGGACAGGAAGAAACT-3′
	*FGFR2*	**cDNA**	R	5′-GTGTGGATACTGAGGAAG-3′
*FGFR2-TACC3*	*FGFR2*	**gDNA**	F	5′-TGACCCCCTAATCTAGTTGC-3′
	*TACC3*	**gDNA**	R	5′-AACCTGTCCATGATCTTCCT-3′

F - forward, R - reverse.

The FGFR genes encode multiple structural variants through alternative splicing. Notably, RNASeq data revealed that the *FGFR2*-IIIb isoform was present in all fusions detected in our study and has been shown to have selectivity for epithelial cells as opposed to the *FGFR2*-IIIc isoform, which is found selectively in mesenchymal cells [81]. Paradoxically, wildtype *FGFR2-IIIb* has been described as a tumor suppressor in pre-clinical systems of bladder cancer and prostate cancer [82], [83]. As such, FGFR signaling appears context-dependent and exhibits variability in disparate tumor types.

Importantly, one critical study has shown that *FGFR2* carboxy-terminal deletion mutants induce ligand-independent transformation and clonogenic growth [84]. This is important because all of the fusion events within our study would lead to loss of the carboxy-terminus of *FGFR2*. Furthermore, a very recent study that described FGFR fusions in solid tumors illustrated that FGFR fusion partners in SIC resulted in dimerization domains, and suggested that activation occurred through ligand independent dimerization and oligomerization [54]. It is likely that both loss of the carboxy terminus and the addition of dimerization domains leads to oncogenic *FGFR2* activity in these tumors.

Comparative pathway analysis of genes carrying mutations/aberrant in copy number identified additional potential therapeutic targets belonging to, or intimately integrated with, the EGFR and FGFR signaling pathways (**Figure 3** **, Tables S2, S3, S4**). Interestingly, most of these pathways also have known roles in mediating epithelial-to-mesenchymal cell transitions, which occur frequently during development as well as during tumorigenesis [60]. Patients 3 and 4 harbored aberrations in several genes acting in cadherin signaling pathways (**Tables S3, S4**), which are important for maintaining cell-cell adhesion [63].

The preliminary anti-tumor activity noted in a patient with *FGFR2-MGEA5* (Patient 4) and *FGFR2-TACC3* fusion (Patient 6) represent the first reports of application of FGFR inhibitors to the treatment of patients with cholangiocarcinoma harboring these alterations. These results suggest that oncogenic activation of *FGFR2* represent a potential therapeutically actionable event. The FGFR tyrosine kinase inhibitors (TKI) dovitinib [85] and NVP-BGJ398 [86] are currently in clinical development and the FGFR TKI ponatinib [75], [87] was recently approved by the FDA for use in treating T315I mutant chronic myelogenous leukemia. *FGF7* (keratinocyte growth factor) has been previously linked to poor prognosis in patients with biliary tract cancer and a small molecule FGFR kinase inhibitor, Ki23057, has demonstrated efficacy in preclinical models [88]. It should be recognized that small molecule tyrosine inhibitors are almost universally promiscuous with regards to specificity and typically significant off-target effects are resultant. Off target efficacy resulting from inhibition of angiogenic kinases in addition to FGFR2 inhibition could explain the anti-tumor activity exhibited in patient 6, as pazopanib has been shown to have nanomolar range potency towards *VEGFR1-3*, *PDGFRA/B* and *CKIT* as well [89].

Larger trials, preferably of a randomized nature with a control arm, need to be conducted to truly define the role of FGFR inhibitors in the treatment of patients with cholangiocarcinoma, particularly those harboring FGFR2 fusions. While our results provide impetus and enthusiasm towards this end, at this stage they should be considered preliminary in nature.

The preliminary anti-tumor activity observed in patient 6 with both pazopanib, and subsequently ponatinib, is particularly intriguing, but also raises important questions. There was an initial response to pazopanib, followed by disease progression. This is a phenomenon observed with the clinical application of most targeted therapeutic approaches. Potential explanations include tumor heterogeneity resulting from clonal selection, transcriptional up-regulation of escape pathways, epigenetic mechanisms and other yet undefined mechanisms of resistance to therapy. The patient did not have additional known alterations in key oncogenic pathways in genes such as *BRAF*, *KRAS*, *EGFR* and *PIK3CA*, which if present, could provide a putative basis for eventual escape from FGFR pathway inhibition. It is unclear why patient 6 initially responded to pazopanib followed by resistance and subsequently responded to ponatinib, another FGFR inhibitor. Putative explanations include the higher potency of ponatinib observed *in vitro* to FGFR2 (IC50≈8 nM for ponatinib vs. 350 nM for pazopanib) and resistance being defined as >20% increase in sum of largest diameters per RECIST v1.1 standard criteria that triggered a discontinuation from pazopanib and recapturing of anti-tumor activity by subsequent inhibition of the FGFR pathway which still maintained therapeutic relevance in that patient at a later time point.

*ERRFI1* has a role as a negative regulator of *EGFR* dependent skin morphogenesis [90], [91], uterine steroid hormone responsiveness [92] and as a tumor suppressor gene [90], [93], [94]. *ERRFI1* is an endogenous inhibitor of *EGFR*, *ERRB2*, *ERRB3* and *ERRB4* through direct interaction with the kinase domains of these proteins [95], [96] and endocytosis/lysosomal degradation of ERBB receptors [97]. *ERRFI1* deletions have been found in glioblastoma multiforme and breast cancer [98]–[100]. Other mechanisms of *ERRFI1* loss include methylation, acetylation and loss of function mutations [98], [101], [102]. Consistent with a driver role of this mutation, previously germline homozygous disruption of *ERRFI1* in mice induces hyperplasia and adenoma formation in the epithelium and development of spontaneous adenocarcinomas of the lung, gallbladder and biliary tract [103]. The tyrosine kinase inhibitor gefitinib has demonstrated anti-tumor activity in mice in spontaneous tumors driven by *ERRFI1* germline loss [91].

Our results suggest immediate and actionable implications for SIC patients with tumors harboring *ERFFI1* loss of function mutations or *FGFR* fusions, given the clinical availability of FDA-approved EGFR and FGFR tyrosine kinase inhibitors. Antibodies specific to *FGFR2*-IIIb have also shown preclinical efficacy and may serve as an additional platform for therapeutic development in this context [104]. Additional studies to characterize the prevalence of these aberrations in both sporadic and liver fluke associated BTC will need to be conducted. Nevertheless, our results suggest that prospective clinical studies designed to treat patient's tumors harboring these novel genomic aberrations utilizing targeted agents on an individualized basis should be pursued more fully through larger clinical studies in order to explore the precise extent of clinical benefit that this tailored approach may have in patients with primary or advanced BTC.

Additionally, post-treatment biopsies to assess pathway down-regulation in patients 4 and 6 (treated with FGFR inhibitors) and patient 3 (treated with EGFR inhibitor) are not available, as the treatment was not conducted in the setting of a protocol that would allow for the collection of additional research biopsies. Incorporation of post-treatment biopsies in carefully designed prospective studies will be critical towards defining the association between the use of FGFR and EGFR inhibitors in appropriately selected patients with relevant genomic aberrations.

## Materials and Methods

### Ethics statement and sample collection

Clinical information was assimilated from patient records from the Mayo Clinic. Informed consent was obtained for each patient on two ongoing research protocols approved by the Mayo Clinic Institutional Review Board (10-006180 and 10-002879). Clinicopathological features collected included age, gender, stage, histological grade, sites of metastasis, tumor sample assessment for overall cellularity/necrosis and percent tumor cellularity, prior therapies and risk factors (hepatitis B and C, Caroli's disease, obesity, hepatolithiasis and cholelithiasis, primary sclerosing cholangitis, thorotrast exposure and *H. pylori, H. bilis, S. typhi* and *S. paratyphi* infections). All patients were known to not have had prior exposure to liver flukes that have been implicated in biliary carcinogenesis (*O. viverrini and C. sinensis*). Tissue specimens were collected fresh frozen and maintained below −80°C until nucleic acid extraction. A board certified pathologist who is experienced in biospecimen studies, evaluated a portion of each specimen to confirm the presence of tumor, the degree of necrosis and the percent cellularity.

### Whole genome sequencing

#### Patients 1, 3, 4, and 5

1.1 µg genomic DNA was used to generate separate long insert whole genome libraries for each sample using Illumina's (San Diego, CA) TruSeq DNA Sample Prep Kit (catalog# FC-121-2001). In summary, genomic DNAs are fragmented to a target size of 900–1000 bp on the Covaris E210. 100 ng of the sample was run on a 1% TAE gel to verify fragmentation. Samples were end repaired and purified with Ampure XP beads using a 1∶1 bead volume to sample volume ratio, and ligated with indexed adapters. Samples are size selected at approximately 1000 bp by running samples on a 1.5% TAE gel and purified using Bio-Rad Freeze ‘n Squeeze columns and Ampure XP beads. Size selected products are then amplified using PCR and products were cleaned using Ampure XP beads.

#### Patient 2

300 ng genomic tumor and normal DNA was used to create whole genome libraries. Samples were fragmented on the Covaris E210 to a target size of 200–300 bp and 50 ng of the fragmented product was run on a 2% TAE gel to verify fragmentation. Whole genome libraries were prepared using Illumina's TruSeq DNA Sample Prep Kit.

### Exome sequencing

#### Patients 1 and 3

1.1 µg genomic DNA for each sample was fragmented to a target size of 150–200 bp on the Covaris E210. 100 ng of fragmented product was run on TAE gel to verify fragmentation. The remaining 1 µg of fragmented DNA was prepared using Agilent's SureSelect^XT^ and SureSelect^XT^ Human All Exon 50 Mb kit (catalog# G7544C).

#### Patient 2

50 ng genomic tumor and normal DNA was used to create exome libraries using Illumina's Nextera Exome Enrichment kit (catalog# FC-121-1204) following the manufacturer's protocol.

#### Patients 4 and 5

1 µg of each tumor and germline DNA sample was used to generate separate exome libraries. Libraries were prepared using Illumina's TruSeq DNA Sample Prep Kit and Exome Enrichment Kit (catalog# FC-121-1008) following the manufacturer's protocols.

#### Patient 6

3 µg of genomic tumor and normal DNA was fragmented on the Covaris E210 to a target size of 150–200 bp. Exome libraries were prepared with Agilent's (Santa Clara, CA) SureSelectXT Human All Exon V4 library preparation kit (catalog# 5190-4632) and SureSelectXT Human All Exon V4+UTRs (catalog# 5190-4637) following the manufacturer's protocols.

### RNA sequencing

#### Patients 1, 2 and 3

50 ng total RNA was used to generate whole transcriptome libraries for RNA sequencing. Using the Nugen Ovation RNA-Seq System v2 (catalog# 7102), total RNA was used to generate double stranded cDNA, which was subsequently amplified using Nugen's SPIA linear amplification process. Amplified products were cleaned using Qiagen's QIAquick PCR Purification Kit and quantitated using Invitrogen's Quant-iT Picogreen. 1 µg of amplified cDNA was fragmented on the Covaris E210 to a target size of 300 bp. Illumina's TruSeq DNA Sample Preparation Kit was used to prepare libraries from 1 µg amplified cDNA.

#### Patients 4, 5 and 6

1 µg of total RNA for each sample was used to generate RNA sequencing libraries using Illumina's TruSeq RNA Sample Prep Kit V2 (catalog# RS-122-2001) following the manufacturer's protocol.

### Paired end sequencing

Libraries with a 1% phiX spike-in were used to generate clusters on HiSeq Paired End v3 flowcells on the Illumina cBot using Illumina's TruSeq PE Cluster Kit v3 (catalog# PE-401-3001). Clustered flowcells were sequenced by synthesis on the Illumina HiSeq 2000 using paired-end technology and Illumina's TruSeq SBS Kit.

### Alignment and variant calling

#### Whole genome and whole exome

For whole genome and exome sequencing fastq files were aligned with BWA 0.6.2 to GRCh37.62 and the SAM output were converted to a sorted BAM file using SAMtools 0.1.18. BAM files were then processed through indel realignment, mark duplicates, and recalibration steps in this order with GATK 1.5 where dpsnp135 was used for known SNPs and 1000 Genomes' ALL.wgs.low_coverage_vqsr.20101123 was used for known indels. Lane level sample BAMs were then merged with Picard 1.65 if they were sequenced across multiple lanes. Comparative variant calling for exome data was conducted with Seurat [105].

Previously described copy number and translocation detection were applied to the whole genome long insert sequencing data [59] and these are made available through https://github.com/davcraig75/tgen_somaticSV. Copy number detection was based on a log2 comparison of normalized physical coverage (or clonal coverage) across tumor and normal whole genome long-insert sequencing data, where physical coverage was calculated by considering the entire region a paired-end fragment spans on the genome, then the coverage at 100 bp intervals was kept. Normal and tumor physical coverage was then normalized, smoothed and filtered for highly repetitive regions prior to calculating the log2 comparison. Translocation detection was based on discordant read evidence in the tumor whole genome sequencing data compared to its corresponding normal data. In order for the structural variant to be called there needs to be greater than 7 read pairs mapping to both sides of the breakpoint. The unique feature of the long-insert whole-genome sequencing was the long overall fragment size (∼1 kb), where by two 100 bp reads flank a region of ∼800 bp. The separation of forward and reverse reads increases the overall probability that the read pairs do not cross the breakpoint and confound mapping.

#### RNA

For RNA sequencing, lane level fastq files were appended together if they were across multiple lanes. These fastq files were then aligned with TopHat 2.0.6 to GRCh37.62 using ensembl.63.genes.gtf as GTF file. Changes in transcript expression were calculated with Cuffdiff 2.0.2. For novel fusion discovery reads were aligned with TopHat-Fusion 2.0.6 [106] (patients 2, 3, 4 and 6). In addition, Chimerascan 0.4.5 [107] was used to detect fusions in patient 1, deFuse 5.0 [108] used in patients 2, 3 and 5 and SnowShoes [109] for patients 2 and 5.

### Somatic mutation validation

Mutations of potential clinical relevance were confirmed in a Clinical Laboratory Improvement Amendments (CLIA) laboratory with Sanger sequencing or quantitative PCR.

### Immunohistochemistry

The immunohistochemistry was performed following the procedures described previously [110]. Briefly, slides were dewaxed, rehydrated and antigen retrieved on-line on the BondMax autostainer (Leica Microsystems, INC Bannockburn, IL). Slides were then subjected to heat-induced epitope retrieval using a proprietary EDTA-based retrieval solution. Endogenous peroxidase was then blocked and slides were incubated with the following antibodies: FGFR2 (BEK, Santa Cruz, catalog# sc-20735), FGFR3 (C-15, Santa Cruz, catalog# sc-123), panAKT (Cell Signaling Technology, catalog# 4685, pAKT (Cell Signaling Technology, catalog# 4060), EGFR (Cell Signaling Technology, catalog# 4267, pEGFR (Cell Signaling Technology, catalog#2234), MAPK/ERK1/2 (Cell Signaling Technology, catalog# 4695), pMAPK/pERK (Cell Signaling Technology, catalog# 4376) and pFRS2 Y436 (Abcam, catalog# ab78195). Sections were visualized using the Polymer Refine Detection kit (Leica) using diaminobenzidine chromogen as substrate.

### Fluorescent in-situ hybridization (FISH)

FISH was performed on formalin-fixed paraffin-embedded (FFPE) specimens using standard protocols and dual-color break-apart rearrangement probes specific to the FGFR2 gene (Abbott Molecular, Inc. Des Plaines, IL) located at 10q26. The 5′ FGFR2 signal was labeled with Spectrum Orange (orange) and the 3′ FGFR2 signal was labeled with Spectrum Green (green).

## Supporting Information

Table S1 Somatic point mutations, insertions and deletions identified in all samples.
(DOCX)

Table S2 Gene Ontology (GO) functional classification of genes carrying Smallscale Nucleotide Variations (SsNVs) or in regions exhibiting Copy Number Variation (CNV).
(XLS)

Table S3 Many genes carrying SsNVs act in key Cancer-associated pathways, and are differentially expressed in tumors from 6 patients with advanced sporadic biliary tract cancer.
(XLS)

Table S4 Many genes identified in genomic regions exhibiting Copy Number Variation (CNV) functionally rank by significance to known Cancer-associated pathways.
(XLS)

Table S5 CLIA validation of somatic mutations with therapeutic relevance in 6 patients with advanced, sporadic biliary tract cancer.
(DOCX)

Table S6 Differential gene expression of fibroblast growth factor receptor pathway family members in 6 patients with advanced sporadic biliary tract cancer.
(DOCX)

Text S1 Supplementary discussion.
(DOCX)
