# Supplementary material for: Integrated Genomic Characterization Reveals Novel, Therapeutically Relevant Drug Targets in FGFR and EGFR Pathways in Sporadic Intrahepatic Cholangiocarcinoma
Source: PLoS Genet. 2014 Feb 13;10(2):e1004135. doi: 10.1371/journal.pgen.1004135 (PMC3923676; doi:10.1371/journal.pgen.1004135)
Supplement: Table S1 — Somatic point mutations, insertions and deletions identified in all samples. (DOCX) [file pgen.1004135.s001.docx]

| **Table S1.** Somatic point mutations, insertions and deletions identified in all samples. | | | | | | | | |
| --- | --- | --- | --- | --- | --- | --- | --- | --- |
| **Gene** | **Chr** | **hg19 position** | **Reference allele** | **Alternate**  **allele** | **Amino acid** | **Functional change** | **dbSNP** | **Patient** |
| *ABCB5* | 7 | 20668394 | A | C | L64F | Missense |  | 4 |
| *ABCC2* | 10 | 101554226 | G | T |  | Splice site donor |  | 5 |
| *ABHD2* | 15 | 89731464 | C | G | L286V | Missense |  | 2 |
| *ABHD2* | 15 | 89731469 | A | T | E287D | Missense |  | 2 |
| *AC005754.1* | 5 | 140536859 | G | A | G428E | Missense |  | 5 |
| *AC102948.2* | 17 | 50939812 | A | C | E21D | Missense | rs12945789 | 6 |
| *ADAMTS7* | 15 | 79058090 | A | G | V1388A | Missense | rs2929158 | 6 |
| *ADRA2B* | 2 | 96780986 | C | CTCCTCCTCT | E301ERRK | Insertion |  | 2 |
| *ADRB3* | 8 | 37823489 | C | T | A167T | Missense |  | 5 |
| *AGBL1* | 15 | 86791075 | G | A | A188T | Missense |  | 4 |
| *AHNAK* | 11 | 62289189 | C | T | E4234K | Missense |  | 2 |
| *AKAP1* | 17 | 55183196 | G | T | R166L | Missense |  | 4 |
| *ANK1* | 8 | 41572571 | C | T | V575M | Missense |  | 2 |
| *ANKRD36* | 2 | 97869931 | A | T | T998S | Missense |  | 2 |
| *ANKS1A* | 6 | 34985417 | T | C | S531P | Missense |  | 6 |
| *APOBR* | 16 | 28507414 | GGGA | G | GE351G | Deletion |  | 6 |
| *APOBR* | 16 | 28507412 | A | C |  | Splice site donor | rs62034315 | 6 |
| *AQP7* | 9 | 33386146 | C | A | V95F | Missense |  | 2 |
| *ARFGEF2* | 20 | 47649660 | G | A | R1761Q | Missense |  | 4 |
| *ARHGEF10* | 8 | 1871220 | T | C | I770T | Missense |  | 3 |
| *ART5* | 11 | 3661586 | T | TGGT | P3PP | Insertion | rs33994425 | 6 |
| *ATN1* | 12 | 7045939 | T | G | H503Q | Missense |  | 4 |
| *ATP6V1H* | 8 | 54742032 | A | T | I93K | Missense |  | 5 |
| *B3GALT2* | 1 | 193149824 | C | A | R290L | Missense |  | 5 |
| *BAP1* | 3 | 52442567 | G | A | R60* | STOP gained |  | 6 |
| *BCAN* | 1 | 156626159 | T | TG |  | Insertion |  | 2 |
| *BEST4* | 1 | 45252249 | G | C | L123V | Missense |  | 5 |
| *BGN* | X | 152772539 | G | A | E269K | Missense |  | 6 |
| *BIN1* | 2 | 127816701 | G | A | S296 | Silent |  | 1 |
| *BOC* | 3 | 112993401 | G | A | A472T | Missense |  | 4 |
| *BSX* | 11 | 122848488 | C | T | G191S | Missense |  | 4 |
| *BTBD7* | 14 | 93709061 | C | T | S986N | Missense |  | 5 |
| *C12orf42* | 12 | 103699824 | C | A | A187S | Missense |  | 5 |
| *C14orf23* | 14 | 29261244 | G | A | G94E | Missense |  | 5 |
| *C16orf89* | 16 | 5115785 | G | A | A80V | Missense |  | 6 |
| *C1orf173* | 1 | 75078454 | G | T | P347H | Missense |  | 5 |
|  |  |  |  |  |  |  |  |  |
| **Table S1 continued.** Somatic point mutations, insertions and deletions identified in all samples. | | | | | | | | |
| **Gene** | **Chr** | **hg19 position** | **Reference allele** | **Alternate**  **allele** | **Amino acid** | **Functional change** | **dbSNP** | **Patient** |
| *C2orf44* | 2 | 24260734 | T | A | Q544L | Missense |  | 5 |
| *C4orf29* | 4 | 128949637 | C | G | S236C | Missense |  | 4 |
| *CABLES2* | 20 | 60967533 | C | A | V335L | Missense |  | 2 |
| *CASQ2* | 1 | 116311087 | G | A | P26S | Missense |  | 5 |
| *CCDC38* | 12 | 96292451 | C | A | R143M | Missense |  | 5 |
| *CCDC40* | 17 | 78011958 | G | C | E22D | Missense |  | 5 |
| *CCDC67* | 11 | 93088628 | C | T | R41W | Missense |  | 2 |
| *CCNA1* | 13 | 37012869 | A | G | E253G | Missense |  | 3 |
| *CDH24* | 14 | 23523858 | T | C | N214S | Missense |  | 4 |
| *CEP128* | 14 | 81244345 | G | A | Q753* | STOP gained |  | 6 |
| *CES5A* | 16 | 55907842 | G | A | P90S | Missense |  | 5 |
| *CLCNKA* | 1 | 16357130 | A | C | K528T | Missense |  | 3 |
| *CNGA3* | 2 | 99013670 | TG | T |  | Deletion |  | 4 |
| *CNTFR* | 9 | 34564827 | G | T | A30E | Missense |  | 6 |
| *COL7A1* | 3 | 48619775 | G | A | P1539L | Missense |  | 3 |
| *CORIN* | 4 | 47663820 | C | A | G548V | Missense |  | 5 |
| *CORO7* | 16 | 4410471 | G | C | P666A | Missense |  | 5 |
| *CPPED1* | 16 | 12798592 | C | T | V202I | Missense |  | 4 |
| *CR1* | 1 | 207785347 | T | A | L2179H | Missense |  | 5 |
| *CSAD* | 12 | 53574520 | G | C | T58S | Missense |  | 4 |
| *CSPG4* | 15 | 75980247 | G | A | D1053 | Silent |  | 1 |
| *CSPG4* | 15 | 75982042 | G | A | T455M | Missense |  | 2 |
| *CTNND1* | 11 | 57582935 | G | A | G924D | Missense |  | 4 |
| *DACT2* | 6 | 168708157 | G | A | I760 | Silent |  | 2 |
| *DCLRE1C* | 10 | 14951028 | T | C | V486 | Silent |  | 1 |
| *DEDD* | 1 | 161094226 | G | T | S9R | Missense |  | 3 |
| *DENND4B* | 1 | 153904906 | C | T | R1257Q | Missense |  | 6 |
| *DHRS13* | 17 | 27225592 | G | T | S334Y | Missense |  | 5 |
| *DIAPH2* | X | 96220118 | C | A | N654K | Missense |  | 6 |
| *DIAPH2* | X | 96220119 | T | A | C655S | Missense |  | 6 |
| *DLGAP4* | 20 | 35060900 | C | A | N260K | Missense |  | 6 |
| *DLL1* | 6 | 170592453 | C | T | A638 | Silent |  | 1 |
| *DMD* | X | 32519893 | G | T | L787M | Missense |  | 5 |
| *DNAH10* | 12 | 124414268 | A | G | R4074G | Missense |  | 5 |
| *DNAH5* | 5 | 13919401 | G | C | L287V | Missense |  | 6 |
| *DNAH5* | 5 | 13920604 | G | C | I261M | Missense |  | 6 |
| *DPEP1* | 16 | 89704048 | G | A | D288N | Missense |  | 1 |
|  |  |  |  |  |  |  |  |  |
| **Table S1 continued.** Somatic point mutations, insertions and deletions identified in all samples. | | | | | | | | |
| **Gene** | **Chr** | **hg19 position** | **Reference allele** | **Alternate**  **allele** | **Amino acid** | **Functional change** | **dbSNP** | **Patient** |
| *DYNC2H1* | 11 | 103036631 | T | C | L1539S | Missense |  | 5 |
| *EDARADD* | 1 | 236645617 | T | A | L106M | Missense |  | 3 |
| *EFCAB5* | 17 | 28380426 | T | TA |  | Insertion |  | 4 |
| *EGR2* | 10 | 64573347 | A | G | F351L | Missense |  | 4 |
| *EHF* | 11 | 34680266 | G | A | R287Q | Missense |  | 4 |
| *EP400NL* | 12 | 132588788 | A | T | S75C | Missense |  | 4 |
| *EPHB3* | 3 | 184299151 | G | C | S948T | Missense |  | 5 |
| *ERRFI1* | 1 | 8073509 | C | A | E384* | STOP gained |  | 3 |
| *EYA4* | 6 | 133827298 | C | A | L422I | Missense |  | 4 |
| *F5* | 1 | 169511553 | C | A | K930N | Missense |  | 5 |
| *FAM123A* | 13 | 25744197 | C | A | D521Y | Missense |  | 2 |
| *FAM123A* | 13 | 25744198 | G | A | S520 | Silent |  | 2 |
| *FAM160B1* | 10 | 116595377 | A | T | I126F | Missense |  | 3 |
| *FAM47A* | X | 34150275 | G | A | P41S | Missense |  | 1 |
| *FAM75A6* | 9 | 43624764 | T | A | K1308M | Missense |  | 5 |
| *FAR2* | 12 | 29446379 | A | G | A15 | Silent |  | 1 |
| *FAT1* | 4 | 187540191 | T | G | T2519P | Missense |  | 2 |
| *FAT3* | 11 | 92085373 | G | A | G32E | Missense |  | 1 |
| *FBXO24* | 7 | 100193313 | G | A | R435Q | Missense |  | 4 |
| *FER1L6* | 8 | 125076689 | A | T | T1144S | Missense |  | 3 |
| *FGFR4* | 5 | 176517136 | C | CGTGT |  | Insertion |  | 4 |
| *FLG* | 1 | 152286125 | C | T | G413R | Missense |  | 3 |
| *FRYL* | 4 | 48542736 | T | C | R1977G | Missense |  | 5 |
| *FUBP1* | 1 | 78435615 | C | A | D69Y | Missense |  | 4 |
| *GALK2* | 15 | 49574257 | T | G | I193R | Missense |  | 5 |
| *GALNTL2* | 3 | 16254193 | C | T | R439C | Missense |  | 4 |
| *GDF11* | 12 | 56143304 | C | T | R288* | STOP gained |  | 2 |
| *GLIS1* | 1 | 54060150 | C | A | A142 | Silent |  | 1 |
| *GOLGA5* | 14 | 93276657 | C | A | Q351K | Missense |  | 5 |
| *GOLGA8J* | 15 | 30382094 | A | T | R169W | Missense |  | 5 |
| *GPR107* | 9 | 132890948 | C | T | F538 | Silent |  | 1 |
| *GPR152* | 11 | 67220171 | G | T | L9M | Missense |  | 2 |
| *GPR45* | 2 | 105859245 | G | T | L310 | Silent |  | 2 |
| *GPR50* | X | 150349559 | A | T | T502S | Missense |  | 6 |
| *GPR50* | X | 150349560 | C | T | T502I | Missense |  | 6 |
| *GPR50* | X | 150349563 | C | A | T503N | Missense |  | 6 |
|  |  |  |  |  |  |  |  |  |
| **Table S1 continued.** Somatic point mutations, insertions and deletions identified in all samples. | | | | | | | | |
| **Gene** | **Chr** | **hg19 position** | **Reference allele** | **Alternate**  **allele** | **Amino acid** | **Functional change** | **dbSNP** | **Patient** |
| *GRIK5* | 19 | 42507570 | C | T | V810M | Missense | rs138512626 | 6 |
| *GRIN3A* | 9 | 104500072 | C | T | A64T | Missense |  | 4 |
| *GRIN3A* | 9 | 104335536 | G | A | R1090C | Missense |  | 5 |
| *GRM2* | 3 | 51747129 | C | T | A364V | Missense |  | 4 |
| *GTF2I* | 7 | 74114738 | G | A | G179R | Missense |  | 5 |
| *HECTD1* | 14 | 31647329 | C | T | R91K | Missense |  | 5 |
| *HECW1* | 7 | 43483862 | T | A | I364N | Missense |  | 5 |
| *HECW2* | 2 | 197183352 | C | A | P754 | Silent |  | 2 |
| *HEXIM1* | 17 | 43226861 | G | C | D102H | Missense |  | 2 |
| *HMCN1* | 1 | 186097351 | C | T | R4278* | STOP gained |  | 2 |
| *HRNR* | 1 | 152187809 | G | C | P2099R | Missense |  | 2 |
| *HS3ST2* | 16 | 22926721 | G | GA |  | Insertion |  | 2 |
| *HSD3B7* | 16 | 30997438 | G | A | G79R | Missense |  | 5 |
| *IDH2* | 15 | 90631839 | T | A | R42W | Missense |  | 1 |
| *IFNA7* | 9 | 21201741 | C | A | A142S | Missense |  | 5 |
| *IGSF9B* | 11 | 133790751 | C | G | A957P | Missense |  | 4 |
| *IQCF1* | 3 | 51929270 | C | T | R85H | Missense |  | 5 |
| *IRX4* | 5 | 1878331 | A | T | W438R | Missense |  | 5 |
| *KDM6B* | 17 | 7752241 | G | T | A879S | Missense |  | 3 |
| *KLHL4* | X | 86887341 | G | A | G486S | Missense |  | 4 |
| *KLKB1* | 4 | 187153319 | G | T | G33C | Missense |  | 5 |
| *KLKB1* | 4 | 187153320 | G | T | G33V | Missense |  | 5 |
| *KRT80* | 12 | 52565206 | C | T | S445 | Silent |  | 2 |
| *KRTAP10-2* | 21 | 45970774 | G | A | P190S | Missense | rs76536096 | 6 |
| *LAMA2* | 6 | 129635848 | G | A | G1154R | Missense |  | 3 |
| *LAMB1* | 7 | 107580617 | A | G | L1217P | Missense |  | 4 |
| *LAMC3* | 9 | 133967086 | A | T | E1559V | Missense |  | 5 |
| *LAMC3* | 9 | 133967087 | G | T | E1559D | Missense |  | 5 |
| *LARP4B* | 10 | 858937 | G | C | R716G | Missense |  | 3 |
| *LBR* | 1 | 225599078 | G | A | L383 | Silent |  | 2 |
| *LDHAL6A* | 11 | 18478344 | CT | C |  | Deletion |  | 6 |
| *LGR5* | 12 | 71976277 | G | A | D532N | Missense |  | 2 |
| *LILRA3* | 19 | 54803016 | C | T | G238S | Missense |  | 4 |
| *LMBRD1* | 6 | 70506725 | T | C | I17V | Missense |  | 3 |
| *LRRC2* | 3 | 46580692 | C | T |  | Splice site acceptor |  | 5 |
| *LRRC41* | 1 | 46751810 | G | C | S240* | STOP gained |  | 4 |
|  |  |  |  |  |  |  |  |  |
| **Table S1 continued.** Somatic point mutations, insertions and deletions identified in all samples. | | | | | | | | |
| **Gene** | **Chr** | **hg19 position** | **Reference allele** | **Alternate**  **allele** | **Amino acid** | **Functional change** | **dbSNP** | **Patient** |
| *LRTM2* | 12 | 1943842 | C | T | R113* | STOP gained |  | 2 |
| *LSM11* | 5 | 157171009 | C | G | S84C | Missense |  | 3 |
| *LTN1* | 21 | 30339205 | AT | A |  | Deletion |  | 2 |
| *LURAP1L* | 9 | 12775875 | G | GCGGCGG | G54GGG | Insertion |  | 6 |
| *MAN1A1* | 6 | 119501505 | G | A | Q603* | STOP gained |  | 3 |
| *MAP1B* | 5 | 71495700 | C | T | T2173M | Missense |  | 5 |
| *MIA3* | 1 | 222828121 | G | T | E409D | Missense |  | 6 |
| *MLL3* | 7 | 151853290 | C | T | V64I | Missense |  | 1 |
| *MLN* | 6 | 33763372 | G | A | P82L | Missense |  | 5 |
| *MSH3* | 5 | 79950745 | C | G | P67A | Missense | rs3045983 | 6 |
| *MUC17* | 7 | 100675961 | A | T | T422S | Missense |  | 5 |
| *MUC4* | 3 | 195511993 | A | G | V2153A | Missense | rs71180963 | 6 |
| *MYBL1* | 8 | 67488452 | G | GT |  | Insertion |  | 4 |
| *MYBPC3* | 11 | 47364188 | G | A | A522V | Missense |  | 5 |
| *MYF6* | 12 | 81101811 | A | G | R105G | Missense |  | 5 |
| *MYH6* | 14 | 23870151 | A | T | S393T | Missense |  | 5 |
| *MYH7* | 14 | 23888715 | C | T | R1282Q | Missense |  | 5 |
| *MYO10* | 5 | 16703153 | C | CCT |  | Insertion |  | 4 |
| *MYO15A* | 17 | 18022463 | T | C | Y117H | Missense |  | 4 |
| *NAV3* | 12 | 78573307 | A | G |  | Splice site acceptor |  | 5 |
| *NAV3* | 12 | 78573308 | G | T |  | Splice site acceptor |  | 5 |
| *NDST1* | 5 | 149924968 | C | T | R689W | Missense |  | 3 |
| *NEK3* | 13 | 52718879 | G | T | L272I | Missense |  | 5 |
| *NFYC* | 1 | 41228581 | G | T | V195F | Missense |  | 5 |
| *NIPA2* | 15 | 23006621 | C | CA | -228? | Insertion |  | 6 |
| *NISCH* | 3 | 52514201 | G | T | G473V | Missense |  | 5 |
| *NLRP12* | 19 | 54313043 | C | T | E624K | Missense |  | 5 |
| *NOB1* | 16 | 69776372 | C | T | A368T | Missense |  | 4 |
| *NOP56* | 20 | 2635161 | A | G | I104V | Missense |  | 6 |
| *NOTCH1* | 9 | 139413210 | G | T | T311N | Missense |  | 4 |
| *NOTCH4* | 6 | 32190372 | G | A | P123S | Missense |  | 4 |
| *NRAS* | 1 | 115258745 | C | G | G13R | Missense |  | 1 |
| *OBSCN* | 1 | 228528249 | C | G | P5820A | Missense |  | 5 |
| *ODF4* | 17 | 8243765 | C | A | S132 | Silent |  | 1 |
| *OGFR* | 20 | 61444610 | G | C | G548A | Missense |  | 2 |
|  |  |  |  |  |  |  |  |  |
| **Table S1 continued.** Somatic point mutations, insertions and deletions identified in all samples. | | | | | | | | |
| **Gene** | **Chr** | **hg19 position** | **Reference allele** | **Alternate**  **allele** | **Amino acid** | **Functional change** | **dbSNP** | **Patient** |
| *OPRK1* | 8 | 54163460 | G | C | D46E | Missense |  | 2 |
| *OR10G7* | 11 | 123909014 | C | T | G232E | Missense |  | 5 |
| *OR11H6* | 14 | 20692082 | C | T | R72W | Missense |  | 4 |
| *OR2AT4* | 11 | 74800284 | G | A | L159F | Missense |  | 5 |
| *OR2L5* | 1 | 248185491 | C | A | A81D | Missense |  | 5 |
| *OR2T12* | 1 | 248458440 | C | A | W147C | Missense |  | 1 |
| *OR2T8* | 1 | 248084704 | C | T | R129* | STOP gained |  | 5 |
| *OR4F17* | 19 | 110796 | A | G | I88V | Missense |  | 1 |
| *OR4F6* | 15 | 102345984 | G | A | R21Q | Missense |  | 3 |
| *OR51F2* | 11 | 4842784 | A | T | S57C | Missense |  | 5 |
| *OR5P2* | 11 | 7818004 | T | TA |  | Insertion |  | 2 |
| *OR6C68* | 12 | 55886823 | G | C | R226T | Missense |  | 5 |
| *ORM2* | 9 | 117094168 | T | C | C167R | Missense |  | 1 |
| *PACS2* | 14 | 105843162 | G | T | D287Y | Missense |  | 1 |
| *PAK1* | 11 | 77051696 | G | A | R371C | Missense |  | 2 |
| *PANX3* | 11 | 124489254 | G | A | R201H | Missense |  | 1 |
| *PBRM1* | 3 | 52621409 | A | T | V1028D | Missense |  | 4 |
| *PCDHA1* | 5 | 140167659 | G | A | R595H | Missense |  | 5 |
| *PCDHA11* | 5 | 140250439 | T | G | V584G | Missense |  | 5 |
| *PCDHA13* | 5 | 140263835 | C | G | A661G | Missense |  | 5 |
| *PCDHA9* | 5 | 140228554 | C | T | S158 | Silent |  | 2 |
| *PCDHB13* | 5 | 140596017 | C | A | F774L | Missense |  | 6 |
| *PCDHGA8* | 5 | 140772598 | A | T | Q73L | Missense |  | 5 |
| *PDCD10* | 3 | 167405482 | C | A |  | Splice site acceptor |  | 5 |
| *PGAP1* | 2 | 197708663 | C | A | W825L | Missense |  | 5 |
| *PICK1* | 22 | 38469049 | G | T | D245Y | Missense |  | 3 |
| *PKHD1L1* | 8 | 110530505 | C | G | H3933Q | Missense |  | 3 |
| *PKHD1L1* | 8 | 110530506 | A | G | T3934A | Missense |  | 3 |
| *PLAC9* | 10 | 81901860 | G | A | P29 | Silent |  | 2 |
| *PLCD3* | 17 | 43192548 | G | T |  | Splice site donor |  | 6 |
| *PLEKHM1* | 17 | 43552664 | C | T | R242Q | Missense |  | 2 |
| *PLXNB3* | X | 153036255 | G | A | E708K | Missense |  | 2 |
| *PLXNB3* | X | 153035871 | G | T | S645I | Missense |  | 5 |
| *PNPLA3* | 22 | 44328804 | C | T | A178V | Missense |  | 5 |
| *POP1* | 8 | 99152343 | G | T | W467L | Missense |  | 5 |
| *PORCN* | X | 48369772 | G | A | V76M | Missense |  | 1 |
|  |  |  |  |  |  |  |  |  |
| **Table S1 continued.** Somatic point mutations, insertions and deletions identified in all samples. | | | | | | | | |
| **Gene** | **Chr** | **hg19 position** | **Reference allele** | **Alternate**  **allele** | **Amino acid** | **Functional change** | **dbSNP** | **Patient** |
| *POTEC* | 18 | 14543031 | C | T | G39S | Missense |  | 5 |
| *PPP3CA* | 4 | 102004355 | G | C | A283G | Missense |  | 3 |
| *PRKD2* | 19 | 47197250 | C | T | P486 | Silent |  | 2 |
| *PRMT5* | 14 | 23393585 | C | T | G365R | Missense |  | 5 |
| *PRR14L* | 22 | 32109983 | G | T | P1281H | Missense |  | 4 |
| *PRRT4* | 7 | 127991604 | C | T | R669H | Missense |  | 6 |
| *PRSS38* | 1 | 228003545 | T | A | I43N | Missense |  | 5 |
| *PTK2* | 8 | 141684462 | G | A | P926S | Missense |  | 6 |
| *PTPDC1* | 9 | 96846825 | G | C | D5H | Missense |  | 4 |
| *PTPRK* | 6 | 128318105 | C | G | W929S | Missense |  | 5 |
| *PTPRK* | 6 | 128643330 | C | A | G117* | STOP gained |  | 5 |
| *PTPRU* | 1 | 29602100 | A | C | S429R | Missense |  | 3 |
| *PTTG2* | 4 | 37962488 | C | G | P145A | Missense |  | 3 |
| *PUS1* | 12 | 132416700 | T | C | V42A | Missense |  | 6 |
| *PVRL1* | 11 | 119549254 | G | A | R101W | Missense |  | 6 |
| *RALGAPB* | 20 | 37128218 | A | G | R228G | Missense |  | 5 |
| *RANBP3* | 19 | 5951471 | G | A | S72L | Missense |  | 2 |
| *RFC2* | 7 | 73646526 | C | T | M325I | Missense |  | 6 |
| *RFX8* | 2 | 102029457 | CCTT | C | QG325R | Deletion |  | 4 |
| *RGPD4* | 2 | 108487305 | G | T | A949S | Missense |  | 5 |
| *RGS14* | 5 | 176794020 | G | A | R156 | Silent |  | 1 |
| *RHBDL1* | 16 | 727855 | T | G | F374V | Missense |  | 4 |
| *RNF123* | 3 | 49740127 | C | A | A564D | Missense |  | 3 |
| *RNF168* | 3 | 196229875 | C | T | R57Q | Missense |  | 1 |
| *RNF8* | 6 | 37336380 | G | T | E121* | STOP gained |  | 4 |
| *RP11-231C14.2* | 16 | 29415047 | T | C | Q26R | Missense |  | 2 |
| *RP11-240B13.2* | 8 | 133044279 | G | T | L506M | Missense |  | 5 |
| *RP11-247L20.2* | 14 | 50792352 | G | C | D159H | Missense |  | 1 |
| *RP4-788L13.1* | 1 | 99771833 | C | T | P520L | Missense |  | 2 |
| *RPS6KA6* | X | 83442827 | C | A | E27D | Missense |  | 5 |
| *RPTN* | 1 | 152127306 | G | A | R757* | STOP gained |  | 5 |
| *RUFY2* | 10 | 70154201 | A | G | Y171H | Missense |  | 5 |
| *RXRG* | 1 | 165370627 | C | T | R422H | Missense |  | 4 |
| *SAMD5* | 6 | 147830203 | C | G | L47V | Missense |  | 2 |
| *SASH3* | X | 128926363 | C | T | P136S | Missense |  | 5 |
| *SCML2* | X | 18283706 | T | C | K316R | Missense |  | 4 |
|  |  |  |  |  |  |  |  |  |
| **Table S1 continued.** Somatic point mutations, insertions and deletions identified in all samples. | | | | | | | | |
| **Gene** | **Chr** | **hg19 position** | **Reference allele** | **Alternate**  **allele** | **Amino acid** | **Functional change** | **dbSNP** | **Patient** |
| *SEMA7A* | 15 | 74703233 | C | T | R578H | Missense |  | 3 |
| *SERPINB12* | 18 | 61234206 | C | A | Q414K | Missense |  | 5 |
| *SHD* | 19 | 4280258 | C | T |  | START GAINED |  | 2 |
| *SI* | 3 | 164735803 | C | T | E1159K | Missense |  | 4 |
| *SIGLEC9* | 19 | 51630343 | C | T | R269C | Missense |  | 5 |
| *SLC12A2* | 5 | 127484493 | A | T | K643N | Missense |  | 5 |
| *SLC12A5* | 20 | 44671933 | T | A | L426Q | Missense |  | 3 |
| *SLC17A2* | 6 | 25918760 | G | T | L202I | Missense |  | 5 |
| *SLC26A1* | 4 | 983623 | G | A | G368 | Silent |  | 2 |
| *SLC26A7* | 8 | 92406205 | T | A | S625T | Missense |  | 5 |
| *SLC26A7* | 8 | 92406206 | C | A | S625* | STOP gained |  | 5 |
| *SLC28A3* | 9 | 86928314 | C | T | S39N | Missense |  | 5 |
| *SLC8A2* | 19 | 47960266 | C | T | G421S | Missense |  | 2 |
| *SLC9A5* | 16 | 67300090 | G | C | R727P | Missense |  | 3 |
| *SMTN* | 22 | 31495079 | C | T | R789* | STOP gained |  | 5 |
| *SNTG2* | 2 | 1094064 | T | A | V98D | Missense |  | 3 |
| *SOST* | 17 | 41832940 | G | A | R138C | Missense |  | 3 |
| *SP2* | 17 | 46000582 | C | A | T438 | Silent |  | 2 |
| *STBD1* | 4 | 77230579 | G | A | C168Y | Missense |  | 5 |
| *STON1* | 2 | 48809567 | C | T | R599C | Missense | rs147440328 | 3 |
| *SUMO4* | 6 | 149721659 | G | A | M44I | Missense |  | 6 |
| *SYT9* | 11 | 7335047 | G | A | V307M | Missense |  | 6 |
| *TAF1B* | 2 | 10074088 | G | T | R581I | Missense |  | 2 |
| *TAF1L* | 9 | 32632950 | C | T | M876I | Missense |  | 5 |
| *TBC1D14* | 4 | 6925259 | C | G | P48R | Missense |  | 5 |
| *TBC1D3B* | 17 | 34500231 | G | T | D84E | Missense |  | 2 |
| *TBC1D9* | 4 | 141555204 | C | <DEL> |  | Deletion |  | 1 |
| *TBX20* | 7 | 35242253 | G | T | P378H | Missense |  | 5 |
| *TBX6* | 16 | 30097857 | C | T | G358E | Missense |  | 4 |
| *TCF25* | 16 | 89940100 | C | G | L9V | Missense |  | 4 |
| *TCP10* | 6 | 167787862 | C | A | A256S | Missense |  | 5 |
| *TDRD7* | 9 | 100193273 | G | C | R89P | Missense |  | 3 |
| *TEAD3* | 6 | 35443196 | T | G | M387L | Missense |  | 1 |
| *TEAD3* | 6 | 35454286 | G | A | P68S | Missense |  | 1 |
| *TESK2* | 1 | 45923355 | C | A | V35L | Missense |  | 5 |
| *TEX2* | 17 | 62232233 | C | T | G974R | Missense |  | 5 |
|  |  |  |  |  |  |  |  |  |
| **Table S1 continued.** Somatic point mutations, insertions and deletions identified in all samples. | | | | | | | | |
| **Gene** | **Chr** | **hg19 position** | **Reference allele** | **Alternate**  **allele** | **Amino acid** | **Functional change** | **dbSNP** | **Patient** |
| *THBS1* | 15 | 39881518 | T | G | F630C | Missense |  | 1 |
| *THSD4* | 15 | 71535273 | T | A | D250E | Missense |  | 6 |
| *TK1* | 17 | 76178740 | G | T | A78D | Missense |  | 5 |
| *TMPO* | 12 | 98921729 | C | G | L22 | Silent |  | 1 |
| *TP53* | 17 | 7577120 | C | T | R273H | Missense |  | 2 |
| *TP53* | 17 | 7578415 | A | T | V172D | Missense |  | 5 |
| *TPH2* | 12 | 72343368 | G | A | G181R | Missense |  | 5 |
| *TRIM50* | 7 | 72732831 | T | A | K239M | Missense |  | 5 |
| *TRNT1* | 3 | 3170825 | C | T | S34F | Missense |  | 6 |
| *TSEN54* | 17 | 73520459 | G | T | R516M | Missense |  | 2 |
| *TTC21B* | 2 | 166810193 | A | ACCCGCTCA |  | Insertion |  | 3 |
| *TTLL4* | 2 | 219612061 | C | G | Q739E | Missense |  | 6 |
| *TUBA1A* | 12 | 49579074 | G | T | P359T | Missense |  | 5 |
| *TULP1* | 6 | 35471413 | G | A | R416C | Missense |  | 5 |
| *UNC13C* | 15 | 54308066 | A | T | E989V | Missense |  | 3 |
| *UNC5B* | 10 | 73051570 | C | A | P559H | Missense |  | 5 |
| *VPS13B* | 8 | 100587961 | A | C | A1700 | Silent |  | 1 |
| *WDR72* | 15 | 54015021 | T | C | I80V | Missense |  | 4 |
| *ZFAT* | 8 | 135621053 | G | T | S235* | STOP gained |  | 3 |
| *ZIM2* | 19 | 57326614 | C | A | E1066* | STOP gained |  | 5 |
| *ZNF136* | 19 | 12298509 | A | T | E439V | Missense |  | 5 |
| *ZNF192* | 6 | 28120107 | G | C | Q240H | Missense |  | 5 |
| *ZNF207* | 17 | 30687633 | G | A | K111 | Silent |  | 2 |
| *ZNF238* | 1 | 244217500 | G | A | D142N | Missense |  | 4 |
| *ZNF282* | 7 | 148921312 | G | A | R530H | Missense |  | 6 |
| *ZNF324* | 19 | 58982553 | C | T | H9Y | Missense |  | 6 |
| *ZNF43* | 19 | 21991313 | G | T | P509H | Missense |  | 5 |
| *ZNF43* | 19 | 21991311 | A | G | Y510H | Missense |  | 5 |
| *ZNF43* | 19 | 21991310 | T | G | Y510S | Missense |  | 5 |
| *ZNF567* | 19 | 37210188 | G | T | A188S | Missense |  | 1 |
| *ZNF567* | 19 | 37210189 | C | T | A188V | Missense |  | 1 |
| *ZNF574* | 19 | 42585046 | A | T | E853V | Missense |  | 5 |
| *ZNF606* | 19 | 58499995 | A | C | M91R | Missense |  | 4 |
| *ZNF629* | 16 | 30794092 | G | A | C519 | Silent |  | 1 |
| *ZNF814* | 19 | 58385748 | G | A | A337V | Missense |  | 4 |
| *ZNF90* | 19 | 20229753 | C | T | R464C | Missense |  | 4 |
|  |  |  |  |  |  |  |  |  |
| **Table S1 continued.** Somatic point mutations, insertions and deletions identified in all samples. | | | | | | | | |
| **Gene** | **Chr** | **hg19 position** | **Reference allele** | **Alternate**  **allele** | **Amino acid** | **Functional change** | **dbSNP** | **Patient** |
| *ZNHIT1* | 7 | 100866043 | G | A | D61N | Missense |  | 1 |
| *ZNRF3* | 22 | 29446008 | G | A | S613 | Silent |  | 1 |
| *ZSCAN2* | 15 | 85164726 | G | A | A434T | Missense |  | 2 |
| Chr, chromosome; dbSNP, NCBI dbSNP Short Genetic Variations database. | | | | | |  |  |  |
