# Supplementary material for: Integrated Genomic Characterization Reveals Novel, Therapeutically Relevant Drug Targets in FGFR and EGFR Pathways in Sporadic Intrahepatic Cholangiocarcinoma
Source: PLoS Genet. 2014 Feb 13;10(2):e1004135. doi: 10.1371/journal.pgen.1004135 (PMC3923676; doi:10.1371/journal.pgen.1004135)
Supplement: Table S5 — CLIA validation of somatic mutations with therapeutic relevance in 6 patients with advanced, sporadic biliary tract cancer. (DOCX) [file pgen.1004135.s005.docx]

| **Table S5.** CLIA validation of clinically relevant somatic mutations and genes with altered gene expression. | | | | |
| --- | --- | --- | --- | --- |
| **Gene** | **Location** | **Mutation** | **CLIA report** | **Patient** |
| *ERRFI1* | chr1:8073509 | C/A | G/T - small percentage of T | 3 |
| *IDH2* | chr15:90631839 | T/A | A/T | 1 |
| *NRAS* | chr1:115258745 | C/G | C/G | 1 |
| *PAK1* | chr11:77051696 | G/A | G/A | 2 |
| *FGFR3* | Overexpressed | Fold change=3.32975 | qPCR fold difference = 2.13 | 4 |
| *FGFR3* | Overexpressed | Fold change=3.58524 | qPCR fold difference = 10.88 | 5 |
