# Supplementary material for: Integrated Genomic Characterization Reveals Novel, Therapeutically Relevant Drug Targets in FGFR and EGFR Pathways in Sporadic Intrahepatic Cholangiocarcinoma
Source: PLoS Genet. 2014 Feb 13;10(2):e1004135. doi: 10.1371/journal.pgen.1004135 (PMC3923676; doi:10.1371/journal.pgen.1004135)
Supplement: Table S6 — Differential gene expression of fibroblast growth factor receptor pathway family members in 6 patients with advanced sporadic biliary tract cancer. (DOCX) [file pgen.1004135.s006.docx]

| **Table S6.** Differential gene expression of fibroblast growth factor receptor pathway family members in 5 patients with advanced sporadic biliary tract cancer. | | |
| --- | --- | --- |
| **Patients** | **FGF Family Member** | **Up/Down Regulated**  **(TumorVs.Normal),**  **Ordered by patient** |
| 5 | *FGFR1* | + |
| 1,5 | *FGFR2* | +/- |
| 1,4,5 | *FGFR3* | +/+/+ |
| 1,3,4,5 | *FGFR4* | +/+/+/+ |
| 1,4,5 | *FGF17* | +/+/+ |
| 1,4* | *FGFBP1* | +/+ |
| 1,4* | *FGF8* | +/+ |
| 1,4,5* | *FGFR1OP* | -/-/- |
| 1,4,5 | *FGFBP3* | -/-/- |
| 1,5 | *FGF2* | -/-/- |
| 1,4,5 | *FGF5* | -/-/- |
| 1,4,5 | *FGF7* | -/-/- |
| 1,4,5 | *FGF9* | -/-/- |
| 1,4 | *FGF10* | -/- |
| 3,5 | *FGF12* | +/- |
| 1,4,5 | *FGF21* | -/-/- |
| **Patients** | **Transcripts that undergo changes in splicing during the E-M Transition** | **Up/Down Regulated (TumorVs.Normal), Ordered by patient** |
| 1,4 | *CTNND1* | -/- |
| 1,4,5 | *CD44* | -/-/- |
| **Patients** | **mRNA splicing factor that regulates the formation of epithelial cell-specific isoforms** | **Up/Down Regulated (TumorVs.Normal), Ordered by patient** |
| 1,4,5 | *ESRP1* | +/+/+ |
| *Not significant (10^-3^ is threshold of significance) | | |
