# Supplementary material for: Integrated Genomic Characterization Reveals Novel, Therapeutically Relevant Drug Targets in FGFR and EGFR Pathways in Sporadic Intrahepatic Cholangiocarcinoma
Source: PLoS Genet. 2014 Feb 13;10(2):e1004135. doi: 10.1371/journal.pgen.1004135 (PMC3923676; doi:10.1371/journal.pgen.1004135)
Supplement: Text S1 — Supplementary discussion. (DOCX) [file pgen.1004135.s007.docx]

**SUPPORTING INFORMATION**

**Integrated Genomic Characterization Reveals Novel, Therapeutically Relevant Drug Targets in FGFR and EGFR Pathways in Sporadic Intrahepatic Cholangiocarcinoma**

Mitesh J. Borad^1,2,4,11^, Mia D. Champion^4,9,11^, Jan B. Egan^4,11^, Winnie S. Liang^3^, Rafael Fonseca^1,2,4^, Alan H. Bryce^1,2,4^, Ann E. McCullough^5^, Michael T. Barrett^2,3^, Katherine Hunt^1^, Maitray D. Patel^6^, Scott W. Young^6^, Joseph M. Collins^6^, Alvin C. Silva^6^, Rachel M. Condjella^2^, Matthew Block^2,4^, Robert R. McWilliams^2,4^, Konstantinos N. Lazaridis^4^, Eric W. Klee^4,9^, Keith C. Bible^7^, Pamela Harris^8^, Gavin R. Oliver^4,9^, Jaysheel D. Bhavsar^4,9^, Asha A. Nair^4,9^, Sumit Middha^4,9^, Yan Asmann^4,9^, Jean-Pierre Kocher^4,9^, Kimberly Schahl^4^, Benjamin R. Kipp^10^, Emily G. Barr Fritcher^10^, Angela Baker^3^, Jessica Aldrich^3^, Ahmet Kurdoglu^3^, Tyler Izatt^3^, Alexis Christoforides^3^, Irene Cherni^3^, Sara Nasser^3^, Rebecca Reiman^3^, Lori Phillips^3^, Jackie McDonald^3^, Jonathan Adkins^3^, Stephen D. Mastrian^3^, Pamela Placek^3^, Aprill T. Watanabe^3^, Janine LoBello^3^, Haiyong Han^3^, Daniel Von Hoff^2,3^, David W. Craig^3,11^, A. Keith Stewart^1,2,4^ & John D. Carpten^3,11^

**AFFILIATIONS:**

1. Division of Hematology/Oncology Mayo Clinic, Scottsdale, AZ, U.S.A.
2. Mayo Clinic Cancer Center, Scottsdale, AZ, U.S.A.
3. Translational Genomics Research Institute, Phoenix, AZ, U.S.A.
4. Center for Individualized Medicine, Mayo Clinic, Rochester, MN, U.S.A.
5. Department of Pathology, Mayo Clinic, Scottsdale, AZ, U.S.A.
6. Department of Radiology, Mayo Clinic, Scottsdale, AZ, U.S.A.
7. Mayo Clinic Cancer Center, Rochester, MN, U.S.A.
8. Investigational Drug Branch, National Cancer Institute, Rockville, MD, U.S.A.
9. Department of Biomedical Statistics and Informatics, Mayo Clinic, Scottsdale, AZ, U.S.A.
10. Department of Laboratory Medicine and Pathology, Mayo Clinic, Rochester, MN, U.S.A.
11. Contributed equally to the work

**Running head:** Novel FGFR and EGFR targets in cholangiocarcinoma

**To whom correspondence should be addressed:**

Mitesh J. Borad: borad.mitesh@mayo.edu

John D. Carpten: jcarpten@tgen.org

**SUPPORTING DISCUSSION**

**Genes With Potential Roles In Therapeutic Sensitivity Or Resistance**

*BAP1* (R60*) presented with a truncating mutation that has been previously reported in skin, but have not been reported in biliary cancers [[1](#_ENREF_1)]. Somatic *BAP1* mutations have been identified in a number of tumor types including: breast, endometrium, eye, kidney, large intestine, lung, ovary, pleura, prostate, skin and urinary tract [[1](#_ENREF_1)]. A deubiquitinating enzyme and possible tumor suppressor, BAP1, plays a critical role in the regulation of chromatin modulation and transcription [[2](#_ENREF_2)]. Furthermore, the loss of BAP1 has been associated with tamoxifen resistance in breast cancer, aggressive and metastatic disease in uveal melanomas [[2](#_ENREF_2)].

A nonsynonymous mutation observed in *PTK2* (P926S) occurs in a region of the gene whose protein product interacts with TGFB1I1 and ARHGEF28 [[3](#_ENREF_3)]. PTK2, also known as focal adhesion kinase (FAK), is a tyrosine kinase involved in the regulation of cell migration, proliferation, adhesion, microtubule stabilization and actin cytoskeleton [[4](#_ENREF_4)]. FAK inhibitors are showing promise in preclinical studies [[5-7](#_ENREF_5)], with several Phase I clinical trials completed. One of the completed trials has reported stable disease in 12% of patients [[8](#_ENREF_8)] and another reported in abstract form stable disease in 33% of patients after two cycles [[9](#_ENREF_9)]. Furthermore, FAK interacts with multiple signaling molecules and in multiple pathways suggesting the possible use of therapeutic treatments directly targeting these interactions or targeting downstream targets of PTK2 such as PI3K or mTOR.

A serine/threonine p21 protein-activated kinase 1 (*PAK1*) gene contains a nonsynonymous (R371C) mutation located in the protein kinase domain. The location of this mutation could potentially lead to loss of the critical protein kinase domain [[10](#_ENREF_10)]. While *PAK1* is expressed in many normal tissues, it is highly-expressed in ovarian, breast and bladder cancers [[11](#_ENREF_11)]. PAK1 plays a role in cell motility, proliferation, survival and death although the ability to therapeutically target PAK1 will require further study by tumor type as breast cancer subpopulations have shown response to PAK1 inhibition while non-small cell lung cancer has proven resistant [[11](#_ENREF_11)]. Interestingly, K5-rTA::tet-KRAS^G12D^ mice wildtype for Pak1, responded to treatment with PAK or MEK inhibitors, but did not respond to AKT inhibitors [[12](#_ENREF_12)]. Thus while the observed mutation in *PAK1* is potentially a therapeutic target, further studies will need to be conducted in cholangiocarcinoma to better assess this possibility.

**Supporting References**

1. Forbes SA, Bindal N, Bamford S, Cole C, Kok CY, et al. (2011) COSMIC: mining complete cancer genomes in the Catalogue of Somatic Mutations in Cancer. Nucleic Acids Res 39: D945-950.

2. Murali R, Wiesner T, Scolyer RA (2013) Tumours associated with BAP1 mutations. Pathology 45: 116-126.

3. UniProt C (2012) Reorganizing the protein space at the Universal Protein Resource (UniProt). Nucleic Acids Res 40: D71-75.

4. Schaller MD (2010) Cellular functions of FAK kinases: insight into molecular mechanisms and novel functions. J Cell Sci 123: 1007-1013.

5. Hao HF, Takaoka M, Bao XH, Wang ZG, Tomono Y, et al. (2012) Oral administration of FAK inhibitor TAE226 inhibits the progression of peritoneal dissemination of colorectal cancer. Biochem Biophys Res Commun 423: 744-749.

6. Golubovskaya VM, Figel S, Ho BT, Johnson CP, Yemma M, et al. (2012) A small molecule focal adhesion kinase (FAK) inhibitor, targeting Y397 site: 1-(2-hydroxyethyl)-3, 5, 7-triaza-1-azoniatricyclo [3.3.1.1(3,7)]decane; bromide effectively inhibits FAK autophosphorylation activity and decreases cancer cell viability, clonogenicity and tumor growth in vivo. Carcinogenesis 33: 1004-1013.

7. Sun H, Pisle S, Gardner ER, Figg WD (2010) Bioluminescent imaging study: FAK inhibitor, PF-562,271, preclinical study in PC3M-luc-C6 local implant and metastasis xenograft models. Cancer Biol Ther 10: 38-43.

8. Infante JR, Camidge DR, Mileshkin LR, Chen EX, Hicks RJ, et al. (2012) Safety, pharmacokinetic, and pharmacodynamic phase I dose-escalation trial of PF-00562271, an inhibitor of focal adhesion kinase, in advanced solid tumors. J Clin Oncol 30: 1527-1533.

9. Jones SF, Shapiro G, Bendell JC, Chen EX, Bedard P, et al. (2011) Phase I study of PF-04554878, a second-generation focal adhesion kinase (FAK) inhibitor, in patients with advanced solid tumors. Journal of Clinical Oncology 29: (suppl;abst 3002).

10. Schwarz JM, Rodelsperger C, Schuelke M, Seelow D (2010) MutationTaster evaluates disease-causing potential of sequence alterations. Nat Methods 7: 575-576.

11. Ong CC, Jubb AM, Zhou W, Haverty PM, Harris AL, et al. (2011) p21-activated kinase 1: PAK'ed with potential. Oncotarget 2: 491-496.

12. Chow HY, Jubb AM, Koch JN, Jaffer ZM, Stepanova D, et al. (2012) p21-Activated kinase 1 is required for efficient tumor formation and progression in a Ras-mediated skin cancer model. Cancer Res 72: 5966-5975.
